# Childhood Maltreatment, Depression, and Suicidal Ideation: Critical Importance of Parental and Peer Emotional Abuse during Developmental Sensitive Periods in Males and Females

**DOI:** 10.3389/fpsyt.2015.00042

**Published:** 2015-03-30

**Authors:** Alaptagin Khan, Hannah C. McCormack, Elizabeth A. Bolger, Cynthia E. McGreenery, Gordana Vitaliano, Ann Polcari, Martin H. Teicher

**Affiliations:** ^1^Developmental Biopsychiatry Research Program, McLean Hospital, Belmont, MA, USA; ^2^Department of Psychiatry, Harvard Medical School, Belmont, MA, USA; ^3^School of Nursing, Northeastern University, Belmont, MA, USA

**Keywords:** depression, childhood maltreatment, suicidal ideation, predictive modeling, developmental sensitive periods, gender differences

## Abstract

**Background:** The adverse childhood experience (ACE) study found that risk for depression increased as a function of number of types of childhood maltreatment, and interpret this as a result of cumulative stress. An alternative hypothesis is that risk depends on type and timing of maltreatment. This will also present as a linear increase, since exposure to more types of abuse increases likelihood of experiencing a critical type of abuse at a critical age.

**Methods:** 560 (223M/337F) young adults (18–25 years) were recruited from the community without regard to diagnosis and balanced to have equal exposure to 0–4 plus types of maltreatment. The Maltreatment and Abuse Chronology of Exposure Scale assessed severity of exposure to 10 types of maltreatment across each year of childhood. Major depressive disorder (MDD) and current symptoms were evaluated by SCID, interview, and self-report. Predictive analytics assessed importance of exposure at each age and evaluated whether exposure at one or two ages was a more important predictor than number, severity, or duration of maltreatment across childhood.

**Results:** The most important predictors of lifetime history of MDD were non-verbal emotional abuse in males and peer emotional abuse (EA) in females at 14 years of age, and these were more important predictors across models than number of types of maltreatment (males: *t*_9_ = 16.39, *p* < 10^-7^; females *t*_9_ = 5.78, *p* < 10^-4^). Suicidal ideation was predicted, in part, by NVEA and peer EA at age 14, but most importantly by parental verbal abuse at age 5 in males and sexual abuse at age 18 in females.

**Conclusion:** This study provides evidence for sensitive exposure periods when maltreatment maximally impacts risk for depression, and provides an alternative interpretation of the ACE study results. These findings fit with emerging neuroimaging evidence for regional sensitivity periods. The presence of sensitive exposure periods has important implications for prevention, preemption, and treatment of MDD.

Childhood maltreatment is a well-recognized risk factor for development of depression and suicidal ideation (1–13). The Adverse Childhood Experience (ACE) Study found that maltreatment-related childhood adversity accounted for 54% of the population attributable risk fraction (PARF) for current episodes of depression and 67% of the PARF for suicide attempts (6, 8).

Another key finding from the ACE study is the essentially linear relationship between number of different types of maltreatment-related childhood adversities an individual experienced and their risk for a host of psychiatric and medical disorders (3, 6, 8, 14–27). Multiplicity of exposure correlates strongly with severity of exposure, which has also been identified as a key determinant of risk (2, 28–31). Some studies also suggest a particularly deleterious role for very early or lengthy exposure (32–37).

An alternative hypothesis is that susceptibility is strongly dependent on type and timing of maltreatment with maximum vulnerability, emerging to a specific type of abuse during a narrow sensitive exposure period. This possibility may result in the appearance of a dose-dependent relationship between number of different types of maltreatment and risk because multiplicity of exposure increases likelihood of experiencing the most deleterious forms of adversity at the most susceptible times. Similarly, lengthy exposure increases the chance of experiencing adversity during a sensitive period. Increasing severity scores reflect either exposure to more types of abuse or increasing frequencies of abuse, which enhances risk of experiencing a critical type of maltreatment at a critical age. Further, severity may be a particularly important global predictor of outcome as exposure outside the sensitive period may also lead to poor outcomes, but may need to be substantially more severe than exposure during the sensitive period.

The strongest support for the importance of type and timing of maltreatment comes from neuroimaging studies looking at the association between maltreatment and brain morphology. First, a number of studies, using unbiased whole brain analyses, have shown that exposure to specific types of maltreatment selectively target sensory systems most involved in perceiving the experience. Choi et al. (38) reported that high levels of exposure to parental verbal abuse most significantly affected the integrity of the arcuate fasciculus that interconnects Broca and Wernicke’s area, whereas visually witnessing domestic violence specifically affected the integrity of the inferior longitudinal fasciculus (39), which interconnects visual and limbic systems. Similarly, Tomoda et al. (40) found that parental verbal abuse was associated with alterations in gray matter volume in superior temporal gyrus/auditory cortex, whereas witnessing domestic violence was associated with reduced gray matter volume and thinning of portions of the visual cortex (41). More recently, Heim et al. (42) found that women reporting histories of childhood sexual abuse had thinning in the genital representation region of somatosensory cortex, whereas women reporting emotional abuse did not.

Second, evidence for relatively brief sensitive periods has emerged in a few studies. Andersen et al. (43) reported in a sample of young women with histories of childhood sexual abuse (CSA) that hippocampal volume was most significantly associated with CSA at 3–5 and 11–13 years of age, whereas mid portion of corpus callosum and prefrontal cortex gray matter volume (GMV) were most susceptible at 9–10 and 14–16 years of age, respectively. Delayed vulnerability of anterior cingulate and insula cortex were also reported by Baker et al. (44). Recent studies have reported that portions of visual cortex appeared to be most susceptible to witnessing domestic violence between 11 and 13 years (41), while the inferior longitudinal fasciculus appeared to be most sensitive to WDV between 7 and 13 years (39). Finally, Pechtel and colleagues in a cross-sectional analysis of a high-risk emotional abuse and neglect sample, followed longitudinally from infancy, found that right amygdala volume was most susceptible to even modest levels of maltreatment at 10–11 years of age, whereas right hippocampus was most susceptible at 7 and 13–14 years (45). Hence, these studies suggest that specific brain regions and pathways are maximally susceptible during brief time periods often spanning 2–3 years.

The picture is murkier regarding type and timing of maltreatment and risk for psychopathology. A few studies have provided evidence that one type of maltreatment may be a stronger risk factor than another for a specific outcome. Teicher et al. (46), Anderson et al. (47) and others provided data suggesting that emotional maltreatment was a greater risk factor for depressive disorder than physical abuse. This finding has been confirmed in a recent meta-analysis which indicated that the odds ratio for depressive disorder was much higher following exposure to emotional abuse (3.06-fold, 95% CI: 2.43–3.85) than following physical abuse (1.54-fold, 95% CI: 1.16–2.04) (2). The situation, however, appears to be reversed in terms of risk for drug abuse (2, 47), which was marginally higher in individuals with histories of physical abuse than emotional abuse and significantly higher in those with physical abuse than neglect (2).

Studies that have assessed the importance of timing of maltreatment and risk for psychopathology have traditionally dichotomized childhood exposure into earlier and later periods. Results, however, from this approach have been inconsistent. Some studies have reported that younger ages of exposure are associated with greater externalizing symptoms (33), whereas others have found this to be true for older ages of exposure (34), and some have reported no differences (48). Schoedl et al. (36) reported that adults who were sexually abused in childhood after the age of 12 were 10 times more likely to develop severe symptoms of post-traumatic stress disorder (PTSD), compared to those who experienced sexual abuse prior to the age of 12. Conversely, depressive symptoms were more severe in individuals reporting sexual abuse before age 12 than in those reporting it after age 12 (36). In contrast, Glod and Teicher (49) reported that PTSD in childhood was associated with very early age of exposure (3 years of age), whereas later exposure (6 years) was associated with psychomotor disturbances associated with depression. Thornberry et al. (50) found that adolescent only (ages 12–17) exposure to physical abuse and neglect significantly increased the odds of delinquency, substance use, and depressive symptoms. More recently, they reported that both early and late exposure periods were harmful, with childhood-limited maltreatment significantly associated with higher levels of depression and suicidality, and adolescent maltreatment associated with higher levels of suicidality, general offending, and substance abuse (35).

However, dichotomizing maltreatment into early versus late (or childhood versus adolescent) exposure periods may be too simplistic. Neuroimaging studies suggest that sensitive periods for the effects of maltreatment may be brief and that there may be both early and late windows of vulnerability, as seen most clearly in studies of hippocampal volume (43, 45). A few studies have assessed the impact of exposure during brief time periods. Interestingly, Kaplow and Widom (34) found that exposure at 3–5 years of age was associated with higher rates of PTSD and depression than exposure at 0–2 years or 6–8 years. Similarly, Dunn et al. (37) found that exposure at 3–5 years of age was more strongly associated with depression and suicidality than exposure at 0–2 or 6–8 years, based on data from the National Longitudinal Study of Adolescents. Hence, these studies provide support for the hypothesis that sensitive periods of risk for psychopathology may be relatively brief and easily missed when comparing broad time frames.

Another concern is that many studies on the impact of maltreatment or early adversity fail to consider the importance of peer victimization. The opinion and action of peers becomes progressively more influential during development, and can easily rival or surpass the influence of parents during adolescence (51–54). Hence, failure to consider the consequences of physical, sexual, or emotional abuse from peers may result in a gross underestimate of the importance of adversity during adolescence. Banny et al. (55), in a 2013 study, underscores this point by showing that overt and relational peer victimization during adolescence mediated the association between childhood maltreatment and depression. In short, childhood maltreatment increased risk for depression in this study, but only indirectly by increasing risk for peer victimization, which in turn had a direct impact on mood (55).

A final consideration is that importance of type and timing of maltreatment may differ between genders. We had previously reported that mid-saggital area of the corpus callosum was smaller in children with histories of maltreatment. However, the primary determinant in males was a history of neglect, whereas sexual abuse was the primary determinant in females (56). Hence, a thorough investigation of the importance of type and timing of maltreatment, and search for sensitive exposure periods may require gender specific analyses.

The Maltreatment and Abuse Chronology of Exposure (MACE) Scale was developed to test hypotheses about sensitive periods by assessing severity of recollected exposure to 10 types of maltreatment, including peer victimization, during each year of childhood (57, 58). A potential problem in identifying sensitive periods is collinearity, as there is generally a strong correlation between degree of recollected exposure to a specific type of abuse at one age and degree of exposure at adjacent ages. This problem can markedly interfere with the interpretation of results using multiple regression analysis or structural equation modeling. An alternative approach is to use data mining or predictive analytical techniques, such as random forest regression (59–61). These modern computationally demanding techniques are well suited to the analysis of highly collinear data sets and can handle models with a very large number of predictor variables (59–61).

In short, detailed MACE information on severity of exposure to 10 types of maltreatment across each year of childhood were analyzed using data mining and predictive analytic techniques to provide a novel and potentially powerful means of identifying sensitive periods when exposure has greatest risk for psychopathology. This approach also provides the ability to compare and contrast the relative importance of exposure to different types of maltreatment across development.

## Materials and Methods

### Subject recruitment

This study was approved by the McLean Hospital Institutional Review board. All subjects provided informed written consent and were screened, recruited, and evaluated using previously described methods (62, 63). Briefly, subjects for this study were recruited by advertisement using the general tag line “*Memories of Childhood*.” Subjects were screened by phone for age, handedness, medications, and general health. Subjects who indicated that they were medically healthy, right handed, unmedicated, and between 18 and 25 years of age were provided with a URL and password to a HIPAA-compliant online enrollment system, which collected detailed information on their life experiences, medical and psychiatric history, developmental history, demographics, and psychiatric symptomatology plus the MACE scale. Subjects were required to be free from neurologic disease or head trauma, resulting in loss of consciousness for more than 5 min or for any duration, if medical evaluation provided evidence of a concussion. Subjects were also excluded who had experienced multiple unrelated forms of adversity including natural disaster, motor vehicle accidents, animal attack, near drowning, house fire, mugging, witnessing or experiencing war, gang violence or murder, riot, or assault with a weapon.

Subjects were selected for evaluation, and the sample enriched to approximately balance number of participants exposed to zero, one, two, three, or four or more types of childhood maltreatment. Subjects were selected without regard to psychiatric history, except for high levels of drug or alcohol use, which were grounds for exclusion. Selecting subjects meeting criteria for a specific disorder could bias results by only including the most severely affected subjects. Conversely, selecting subjects without any psychiatric history could bias results in the opposite direction. Subjects unexposed to maltreatment were selected using the same criteria, and were not filtered to be free of psychopathology. Hence, incidence of psychopathology in subjects exposed to zero, one, two, three or four, or more forms of maltreatment should be reflective of incidence rates found in the community. Subjects were paid $25 for completing the online assessment, $100 per interview and assessment session (typically one 4-h sessions), and $100 for a 1 h MRI protocol (results to be published separately).

### Subject assessments

Structured clinical interviews for DSM-IV Axis I and II psychiatric disorders were used for diagnoses. Mental health professionals (psychiatrists, Ph.D. psychologists, clinical nurse specialists) conducted the assessment and evaluation interviews.

Childhood maltreatment was assessed using the MACE Scale. The scale was developed using item response theory and provides excellent overall reliability (*r* = 0.91, *n* = 75), and good to excellent reliability at each age and to each type of maltreatment. MACE-MULTI score indicates the number of different types of childhood adversities experienced, whereas the MACE-SUM score indicates overall severity of exposure. MACE-MULTI score correlated *r* = 0.70 (95% CI: 0.68–0.74, df = 1043, *p* < 10^-16^) with the ACE score (26), while MACE-SUM correlated *r* = 0.74 (95% CI: 0.69–0.78, df = 395, *p* < 10^-16^) with the childhood trauma questionnaire (CTQ) (64) total score. However, MACE-MULTI and MACE-SUM accounted, on average, for 2-fold more of the variance in psychiatric symptom ratings (anxiety, depression, somatization, anger-hostility, dissociation, limbic irritability, suicidality) than ACE or CTQ based on variance decomposition analysis. Hence, this instrument provides the fine grain temporal resolution necessary to identify sensitive periods and to compare potential consequences of exposure to multiple types of maltreatment, albeit, based on retrospective report.

In addition to the MACE, maltreatment was assessed using the 100-item semistructured Traumatic Antecedents Interview (65). This interview evaluates reports of physical and sexual abuse, emotional and physical neglect, witnessing violence, significant separations or loss, verbal abuse and parental discord (65). The reliability of the Traumatic Antecedents Interview variables ranges from acceptable to excellent (65). Subjects were also evaluated using both self-report and interview versions of the conflict-tactic Scales (66), the Childhood Trauma Questionnaire (67, 68), and the Adverse Childhood Experience (16) score.

Low income and poverty are additional important risk factors for both maltreatment and depression. Young adult subjects were often uncertain about parental income while they were growing up. However, they were well aware of the degree of perceived financial sufficiency, or stress they experienced during this time. This was rated from 1 (much less than enough money for our needs) to 5 (much more than enough money for our needs). Perceived financial sufficiency explained a greater share of the variance in symptom ratings than combined family income. Financial sufficiency and parental education were included as two components of socioeconomic status.

Symptoms of depression were assessed using a variety of instruments. The Kellner symptom questionnaire (SQ) was used to provide self-report ratings of psychiatric symptom severity in four domains (depression, anxiety, anger-hostility, somatization). This is a 92-item yes/no questionnaire that provides current ratings during the past week (69). The symptom subscales have excellent test–retest reliability (e.g., depression *r* = 0.95) and correlate well with Hamilton Depression Rating Scale (HDRS) and Symptom Checklist 90 (SCL–90) ratings (69). We have previously found that SQ depression scores were substantially increased in individuals reporting exposure to physical, sexual, and emotional abuse (46), as well as peer verbal abuse (52).

Current week self-report symptoms of depression were also assessed using the SCL–90. The SCL-90, and nearly identical SCL-90-revised (which differs only by the substitution of two anxiety items), provide a widely used 90-item measure of general psychiatric distress comprised of nine subscales (somatization, obsessive–compulsive, interpersonal sensitivity, depression, anxiety, anger–hostility, phobic anxiety, paranoid ideation, and psychoticism) (70, 71). Psychometric evaluations have reported good internal consistency, good test–retest reliability, good concurrent, construct, and discriminant validity (72, 73). Further, reanalysis using item response theory found most of the subscales to be robust (74).

The Adult Suicidal Ideation Questionnaire (75) (ASIQ) was used to assess suicidal ideation during the past month. It consists of 25 items rated on their frequency of occurrence. The scale has high internal consistency (*r*_α_ = 0.97), excellent test–retest reliability (*r* = 0.86), and correlates to a moderate degree (r_s_ = 0.38–0.60) with measures of depression, hopelessness, anxiety, and self-esteem.

The Structured Interview Guide to the Hamilton Depression Scale (76) with Seasonal Addendum (SIGH-SAD) (77) was used to obtain interviewer based ratings of depressive severity. The original 17-item HDRS (78) and 21-item variant have served for decades as the “gold-standard” for measuring severity of depression in clinical trials (79). The HDRS-17 has adequate internal reliability and adequate convergent and discriminant validity. However test–retest for many items are poor, some items contribute only minimally to severity scores, and other items are poorly weighted, raising questions about its continued use as a *de facto* standard (80). Eight items of the 29-item SIGH-SAD HDRS, which measure atypical symptoms (e.g., increased appetite, hypersomnia, carbohydrate craving), were also used as an Atypical-Index.

For contrast we also analyzed symptoms of ‘limbic irritability’, as assessed using the limbic system checklist-33 (LSCL-33) (81), for presence of sensitive exposure periods. We did so because ‘limbic irritability’ is by far the symptom cluster most strongly affected by exposure to childhood maltreatment in our studies (46, 52, 82). Hence, we hypothesized that ‘limbic irritability’ would have a much longer sensitive exposure period than depression ratings, or would be sensitive throughout development resulting in a stronger association with global exposure measures than with exposure to a specific type of maltreatment at a specific age.

Briefly, the LSCL-33 was created to evaluate the frequency with which subjects experience symptoms often encountered as ictal temporal lobe epilepsy phenomena, as described by Spiers et al. (83). These phenomena consist of paroxysmal somatic disturbances, brief hallucinatory events, visual distortions, automatism, and dissociative experiences. Psychometric studies showed that the Limbic System Checklist-33 has high test–retest reliability (*r* = 0.92, *N* = 16) (13). Scores are low in normal comparison subjects (<10) and higher in patients with documented temporal lobe epilepsy (>23). We previously found a substantial association between LSCL-33 scores and blood flow to the cerebellar vermis (84), which through the fastigial nucleus modulates electrical activity within the limbic system (85), and with reduced integrity of the pathway interconnecting visual system and limbic system (39). Limbic system irritability was also found to mediate the risk between childhood maltreatment and symptoms of depression and dissociation (86).

Symptom ratings were not conducted at the same time. The SQ and LSCL-33 were completed online along with the MACE, and the MACE was used to select subjects for interview. Most of the ASIQ ratings were also completed online. The SCID, HDRS, and SCL-90 were completed weeks to months later when subjects were scheduled and arrived for interviews. Hence, SQ and SCL-90, which were used to assess current week symptoms of depression, would not be expected to correlate as strongly as they would have had they been completed during the same session.

### Data analysis

Statistical analyses were conducted in R (version 3.1.0) (87). Logistic regression and multiple regression models were used to confirm previously reported associations between number of types of maltreatment-related adversity experienced during childhood and history of major depressive disorder (MDD) or symptom ratings. Age, gender, perceived financial sufficiency, and parental education were used as covariates.

Linear mixed effect models (R packages *LME4* and *LMERConvenienceFunctions*) were used to determine whether maltreated individuals with history of MDD differed from maltreated individuals without MDD in severity of exposure or timing of exposure to each type of maltreatment. For these analyses, data were covaried by age, sex, and parental education. Subjects were nested within levels of financial sufficiency during childhood.

#### Mediation

Mediation analyses were used to ascertain the degree to which severity of exposure to a specific type of maltreatment during a sensitive exposure period mediated the relationship between number of types of childhood maltreatment experienced and psychopathology. In the classic single variable mediation model, the total effect of the independent variable (IV) on the dependent variable (DV) (path *c*) is mediated indirectly through the mediator (M) via paths *a* (IV > M) and *b* (*M* > *DV*), and directly through path *c*’. Traditionally, mediation is detected through the causal steps approach popularized by Baron and Kenny (88), and/or by the Sobel test (89) to evaluate the significance of the indirect *ab* path coefficient (90). The causal step approach has recently been criticized because simulation studies have shown that this approach is amongst the least powerful method for testing intervening variable effects (91, 92). The Sobel test also has a significant flaw. It requires the sampling distribution of the indirect effect *ab* to be normal, though it tends to be asymmetric with non-zero skew and kurtosis (90, 93). Simulation research shows that modern bootstrap-based methods are more powerful than the Sobel test and the causal steps approach (94, 95). Bootstrapping methods were implemented in R (“*mediation”* in R package *MBESS*) (96) to calculate *a*, *b*, *c*, and *c’*, with *p* values, the indirect effect (*ab*) with 95% confidence intervals, and the ratio of indirect to direct effect (89).

##### Random forest regression with conditional trees

The MACE provides retrospective data on exposure to 10 types of maltreatment across 18 years of development. Our primary interest is whether severity of exposure to a particular type of maltreatment at a specific age is a particularly important risk factor or predictor for developing major depression or symptoms of depression. Conventional analytical techniques such as multiple regression are not suitable for this task, as there is substantial collinearity (or multicollinearity) in degree of exposure to a particular type of maltreatment at adjacent ages.

This situation, however, is a common occurrence in data mining and “big data” analytics, and several techniques exist for identifying important predictor variables under these circumstances. A general strategy is to use predictive modeling in which a machine learning algorithm is trained to provide an accurate fit to a training set, which is then evaluated for predictive validity on a separate test set. Once a good predictive model is established, a variety of techniques exist for identifying the most important predictor variables within the model. This basic strategy has been used successfully in ecological studies to identify features found across different environmental niches that are associated with specific outcomes, such as the population density of a specific species within a niche (97–99).

A particularly useful machine learning strategy is random forest regression, which uses decision trees as the base learners (59). Decision trees by themselves often fit the data well but are typically weak predictors. Random forest regression improves predictive validity by creating a forest of decision trees. Trees within the forest differ from each other, as they are each generated from a different subset of the data, and each tree is constrained in the number of predictor variables it can consider at each decision point (59). New data are run through each tree in the forest and the outcome of the trees averaged.

This counterintuitive “wisdom of the crowd” strategy works well, and generally provides predictive models that are superior to those produced using conventional regression techniques, and are on par with other machine learning approaches such as neural networks and support vector machines (60). In addition to high predictive accuracy, random forests can successfully identify important predictor variables in situations where number of predictors greatly exceeds number of subjects. Further, it does not require that the variables be normally distributed, they can be distributed or scaled in any way, and it is resistant to multicollinearity (59). Random forest regression is also advantageous as the tree structure allows for the detection and modeling of interactive effects between the variables.

Brieman (59) in developing this strategy also provided a novel means of determining variable importance. The importance of each predictor variable is assessed by randomly permuting each variable in turn and determining how much this degrades model fit. Permuting important predictor variables decreases fit to a large degree, whereas permuting unimportant predictors has little or no impact.

We used a variant of Brieman’s approach with conditional trees as the base learner (“*cforest*” in R package *party* (100) called through the *caret* (101) package for predictive modeling). This approach rectifies a potential problem with random forest regression that can inflate the importance of predictor variables with many versus few levels or categories. Random forest with conditional trees appears to provide an unbiased estimate of variable importance that is not influenced by number of categories, mean value, range, or variance of the predictor variables (100).

Training and testing were accomplished using 10 × 10 repetitions of leave group out cross validation (LGOCV) (or Monte Carlo cross validation) (101). Briefly, data were randomly split into training and test sets, with 75% of subjects used to train the model, and 25% used to test the models’ predictive accuracy. Ten runs of LGOCV were performed to provide mean estimates of model fit and variable importance. This process was then repeated 10 times on different random spits to establish confidence limit on the estimates. Data were limited to ages and types of maltreatment that were experienced by at least 5% of the gender-specific sample. For easier visualization, results were collapsed across age or maltreatment type to indicate the maximal importance of exposure to each type of abuse regardless of age, and maximum sensitivity to maltreatment at each age regardless of type of exposure.

Random forest with conditional trees was selected as the optimal machine learning strategy for determining importance of exposure at each age based on simulation studies with artificial outcome data. Actual exposure data (*n* = 560) were used as predictors but outcomes were explicitly calculated from the exposure data to correspond to exposure to one or more types of abuse at specific ages, and then diluted with random noise, so that the type and timing of maltreatment accounted for about 10% of the variance. Simulated data were analyzed using artificial neural networks, general linear model, gradient boosted machines, multiple adaptive regression splines (MARS), partial least squares, random forest regression, and random forest regression with conditional trees. Random forest regression with conditional trees accurately identified the type and timing of exposure used to generate the artificial data in all simulations. Gradient boosted machines also performed well but showed some bias to overinflate importance of predictors with many gradations. MARS tended to identify single predictors in instances when the simulated data were based on multiple adjacent predictors. The other algorithms were moderately to severely influenced by collinearity, identifying ages and types of maltreatment as important predictors when they were not predictors, but correlated with the actual predictors.

Software for conducting these predictive analytical models with MACE scores and for interpreting and graphing results was written in R by the senior author (MT) and are available on request.

##### Statistical criteria for sensitive exposure period

We propose that a sensitive exposure period can be said to exist if exposure to maltreatment during a specific developmental stage is a more important predictor of outcome than overall measures of exposure as indexed by severity of exposure, duration of exposure, and number of different types of adversity (multiplicity of exposure) experienced throughout childhood. Hence, we set as a null hypothesis that sensitive exposure periods were not present in a data set if importance of overall exposure to a given outcome was of equal or greater importance than exposure at one or two adjacent ages. Conversely, the null hypothesis would be rejected, and presence of a sensitive exposure period confirmed if importance of exposure to a specific type of maltreatment at one or two adjacent ages was a significantly more important predictor of outcome than all three overall exposure measures (duration, severity, and multiplicity of exposure).

## Results

### Sample characteristics

The sample consisted of 560 subjects (223M/337F), 22.8 ± 2.1 years of age. Racial breakdown was 69% White, 15% Asian, 9%, Black, 2% American-Indian or Alaskan Native, and 5% other. Ten percent were of Hispanic ethnicity. On average, subjects had completed 15.1 ± 1.8 years of education and many were still in college. Their parents had completed a mean of 15.7 ± 3.0 years. Altogether, 3% indicated that during their childhood their families had much less than enough money, 17% had less than enough money, 46% had enough money, 30% had more than enough money, and 3% had much more than enough money to meet their needs.

### Maltreatment and risk for major depression

As expected, there was a strong association between self-reported exposure to maltreatment and history of MDD. Percent of subjects with lifetime history of MDD in the unexposed group (MACE-MULTI = 0) was 19.2 versus 39.7% in subjects exposed to one or more forms of maltreatment (Fisher exact: *p* < 10^-5^, odds ratio = 2.76, 95% CI: 1.73–4.52). There was also a progressive increase in risk for MDD with exposure to more types of maltreatment. Logistic regression analysis revealed a highly significant relationship between number of types of maltreatment and history of MDD (χ^2^ = 31.22, df = 4, *p* = 10^-5^) that was not significantly affected by age, gender, financial sufficiency, or parental education.

### Maltreatment and symptom severity

**Figure 1:**
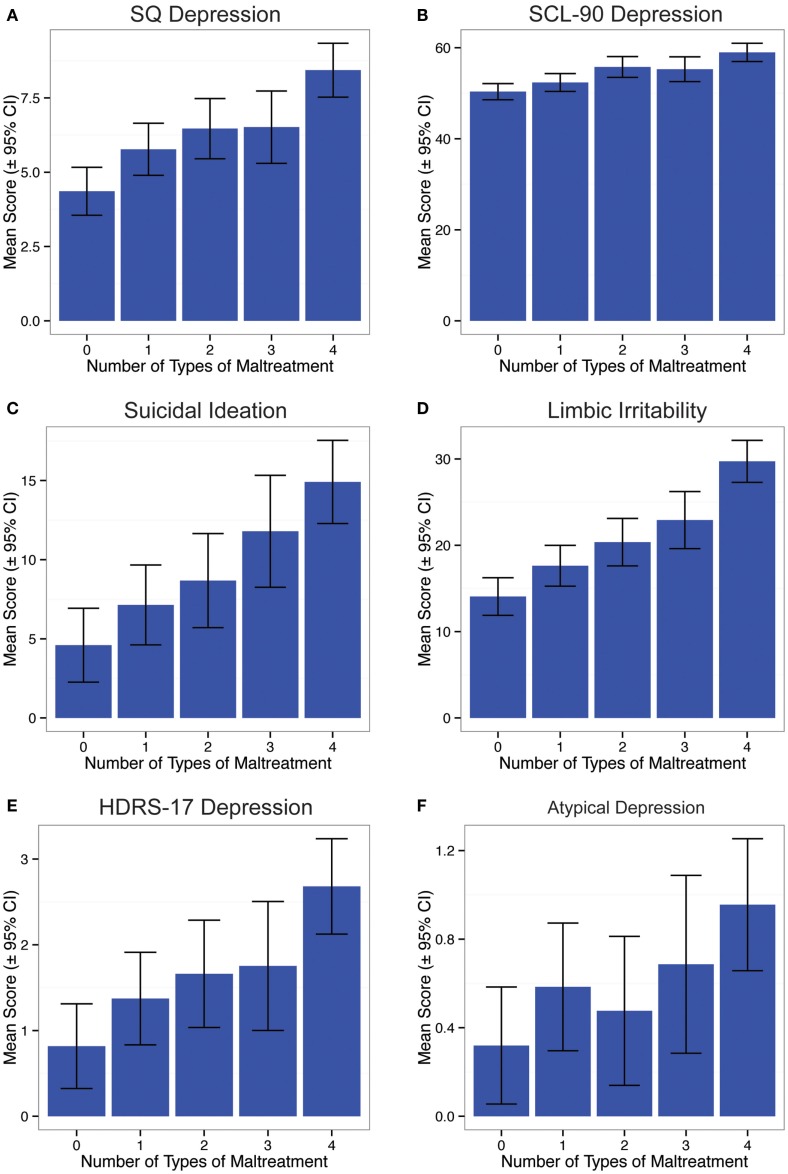
**Relationship between number of different types of maltreatment experienced during childhood and (A) current rating of depression on Kellner’s Symptom Questionnaire, (B) current rating of depression on Symptom Checklist 90, (C) suicidal ideation on the Adult Suicidal Ideation Questionnaire, (D) limbic irritability of the Limbic System Checklist-33, (E) interviewer based depression ratings on Hamilton Depression Rating Scales, and (F) atypical depression rating on SIGH-SAD**.

Ratings of depression and suicidal ideation, covaried for age, gender, parental education, and childhood financial sufficiency, were strongly associated with number of different types of maltreatment subjects reported to have experienced during childhood, as showed the expected linear relationship between outcome and multiplicity of exposure (Figure 1). Main effects of multiplicity of exposure (grouped into scores of 0, 1, 2, 3, and 4 plus) on Kellner’s SQ Depression (F_4,546_ = 10.66, *p* < 10^-7^), SCL- 90 Depression (F_4,539_ = 10.71, *p* < 10^-7^), LSCL-33 (F_4,541_ = 23.55, *p* < 10^-16^), ASIQ (F_4,538_ = 9.71, *p* < 10^-6^), and HDRS-17 (F_4,543_ = 6.35, *p* < 10^-4^) were all highly significant. Main effect of multiplicity of exposure on the SIGH-SAD atypical index was also significant but less robust (F_4,541_ = 2.58, *p* < 0.04).

### Difference in exposure patterns between maltreated individuals with or without histories of major depression

**Figure 2:**
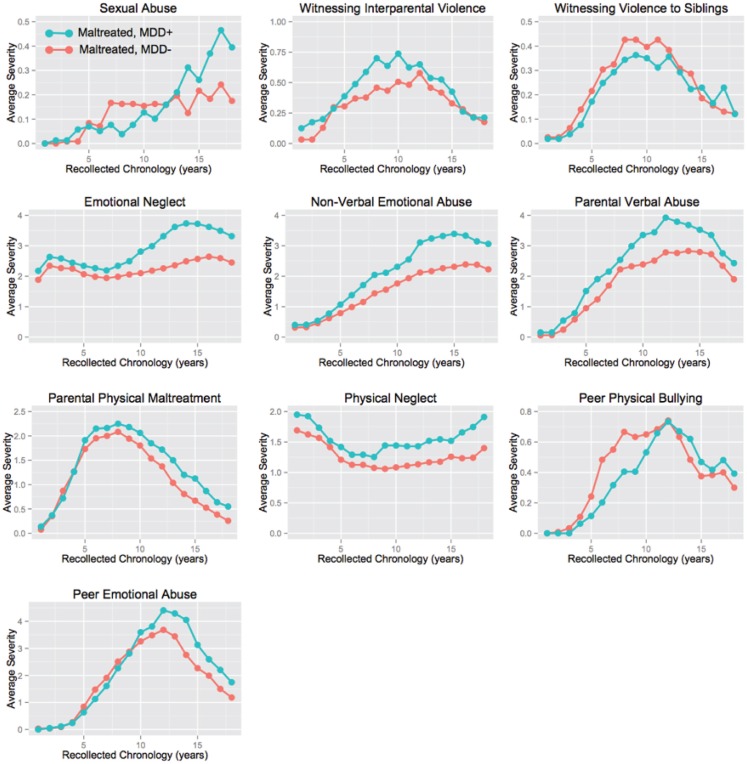
**Time course of exposure to 10 types of abuse or neglect in maltreated subjects with and without major depressive disorder (MDD) history**.

As indicated in Table 1 and Figure 2, linear mixed effects models revealed that maltreated subjects (*n* = 416) with histories of major depression (40%) versus those without (60%) differed significantly in degree of exposure to six types of maltreatment, and differed in time course of exposure on four types. The most significant differences in overall severity of exposure were for non-verbal emotional abuse (F_1,7031_ = 184.47.98, *p* < 10^-40^), emotional neglect (F_1,7031_ = 118.53, *p* < 10^-26^), and parental verbal abuse (F_1,7031_ = 60.38, *p* < 10^-14^). Significant differences in time course in subjects with and without histories of MDD (age × history MDD interaction) were found on ratings of non-verbal emotional abuse (F_17,7031_ = 4.50, *p* < 10^-8^), peer emotional abuse (F_17,7031_ = 3.00, *p* < 10^-4^), emotional neglect (F_17,7031_ = 2.31, *p* < 0.002), and sexual abuse (F_17,7013_ = 1.77, *p* < 0.03). Overall, maltreated individuals with histories of MDD differed most strongly from maltreated subjects without MDD in their greater exposure to parental and peer emotional abuse and parental emotional neglect during adolescence.

**Table 1:** **Mixed linear effects models indicating differences in severity and timing of exposure to 10 types of maltreatment in maltreated individuals with and without major depressive disorder (MDD) history**.

Variable	*F*	*p* value	% Variance	Variable	*F*	*p* value	% Variance
**Sexual abuse**				**Parental physical abuse**			
Age	7.71	<10^−18^	1.8	Age	47.28	<150^−150^	9.94
History MDD	3.03	0.08	0.04	History MDD	9.26	0.002	0.11
Parental ed. (years)	7.75	0.005	0.11	Parental ed. (years)	93.84	<10^−21^	1.16
Gender	38.46	<10^−9^	0.53	Gender	30.85	<10^−7^	0.38
Age × Hx MDD	1.77	0.03	0.41	Age × Hx MDD	0.62	0.88	0.13
**Parental verbal abuse**				**Non-verbal emotional abuse**			
Age	66.58	<10^−211^	13.2	Age	124.22	<10^−308^	20.55
History MDD	60.38	<10^−14^	0.7	History MDD	184.47	<10^−40^	1.79
Parental ed. (years)	72.71	<10^−16^	0.85	Parental ed. (years)	21.36	<10^−5^	0.21
Gender	0.00	0.96	0	Gender	16.22	<10^−4^	0.16
Age × Hx MDD	1.32	0.17	0.26	Age × Hx MDD	4.50	<10^−8^	0.74
**Witnessing interparental Violence**				**Witnessing violence to siblings**			
Age	7.15	<10^−16^	1.63	Age	8.89	<10^−22^	2.06
History MDD	3.20	0.07	0.04	History MDD	2.88	0.09	0.04
Parental ed. (years)	87.34	<10^−19^	1.17	Parental ed. (years)	109.16	<10^−24^	1.49
Gender	0.24	0.62	0	Gender	0.44	0.51	0.01
Age × Hx MDD	0.31	0.99	0.07	Age × Hx MDD	0.28	0.99	0.07
**Emotional neglect**				**Physical neglect**			
Age	7.57	<10^−18^	1.69	Age	3.99	<10^−7^	0.89
History MDD	118.53	<10^−26^	1.56	History MDD	21.38	<10^−5^	0.28
Parental ed. (years)	67.14	<10^−15^	0.88	Parental ed. (years)	97.11	<10^−22^	1.27
Gender	2.59	0.11	0.03	Gender	1.27	0.26	0.02
Age × Hx MDD	2.31	0.002	0.52	Age × Hx MDD	0.30	0.99	0.07
**Peer emotional abuse**				**Peer physical abuse**			
Age	107.30	<10^−308^	20.05	Age	16.38	<10^−47^	3.69
History MDD	16.36	<10^−4^	0.18	History MDD	2.09	0.15	0.03
Parental ed. (years)	28.07	<10^−6^	0.31	Parental ed. (years)	2.20	0.14	0.03
Gender	123.39	<10^−27^	1.36	Gender	213.58	<10^−46^	2.83
Age × Hx MDD	3.00	<10^−4^	0.56	Age × Hx MDD	0.98	0.48	0.22

### Type and timing of maltreatment and risk for major depressive disorder

#### Males

The random forest classification with conditional trees model showed significant predictive ability, based on leave group out cross-validation (LGOCV), with predictive accuracy of 0.719 ± 0.051, kappa = 0.202 ± 0.153 (t_9_ = 4.17, *p* < 0.002), and receiver operating characteristic area (ROC-area) of 0.586 ± 0.067 (t_9_ = 4.09, *p* = 0.002). Note, these values do not indicate the fit between the model and the raw data, which is much higher. Rather, they indicate the average predictive fit of models trained on 75% of the subjects when applied to the remaining 25%. Average fit across models on test and training set together provided specificity of 0.980 (95% CI: 0.943–0.996), sensitivity of 0.439 (95% CI: 0.317–0.567), and kappa = 0.490 (95% CI: 0.364–0.617). Altogether, subjects predicted to have MDD according to the model had a 38.4-fold (95% CI: 11.1–133.0) increase in odds of actually having a history of MDD.

**Figure 3:**
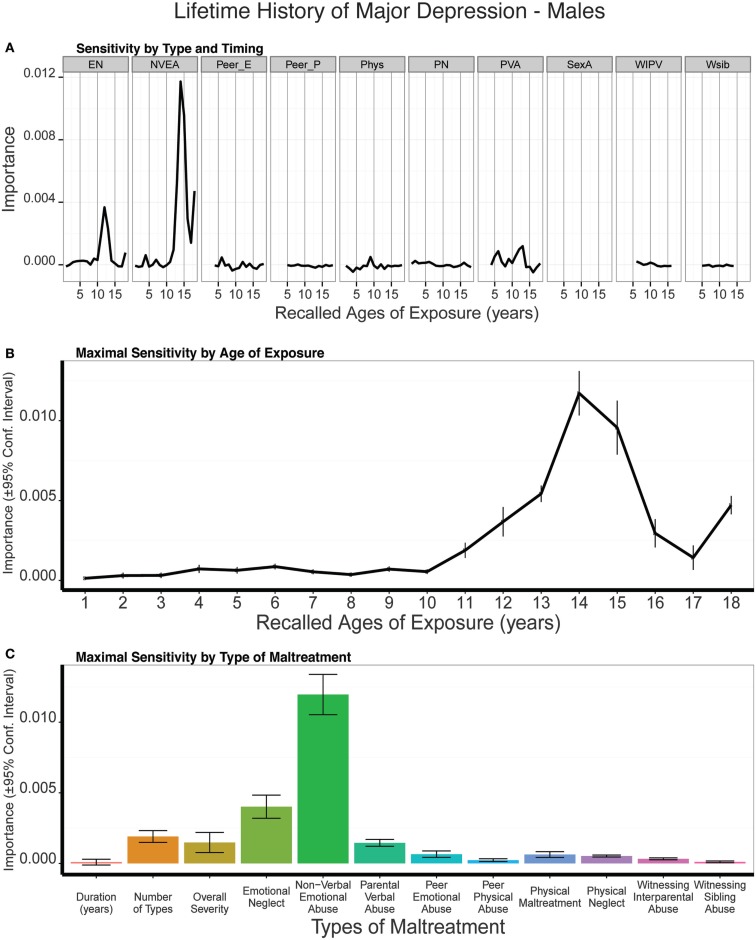
**(A)** Mean importance of age of exposure for each type of maltreatment in predicting history of major depressive disorder in males. Values are missing for ages of exposure for some types of maltreatment if <5% of subjects reported exposure at that age. **(B)** Maximal importance of age of exposure (regardless of type) and **(C)** maximal importance of type of maltreatment (regardless of age) in predicting history of major depressive disorder in males. Abbreviations: EN = emotional neglect, NVEA = non-verbal emotional abuse, Peer E = peer emotional maltreatment, Peer P = peer physical maltreatment, PN = physical neglect, PVA = parental verbal abuse, SexA = sexual abuse, WIPV = witnessing interparental violence, Wsib = witnessing violence to siblings.

Figure 3A shows the mean importance of age of exposure for the 10 types of maltreatment. To better understand these results, we also collapsed the data to show: (1) maximal sensitivity across development regardless of type of abuse (Figure 3B); and (2) maximal sensitivity to type of abuse (regardless of age) versus global exposure measures (Figure 3C). Exposure to non-verbal emotional abuse at 14 years was consistently (across the 10 LGOCV iterations) the most important single predictor in males, and this was a much more important predictor than duration (*t*_9_ = 21.93, *p* < 10^-8^), multiplicity (*t*_9_ = 16.39, *p* < 10^-7^), or severity (*t*_9_ = 16.263, *p* < 10^-7^) of exposure. Exposure to emotional neglect at age 12 was the second most important predictor, which was also a more important single predictor than the global exposure measures. Together, peak sensitivity to emotional neglect at age 12 and non-verbal emotional abuse at age 14 resulted in a composite sensitivity profile characterized by an emerging increase in sensitivity at age 11, peak sensitivity at age 14, and sharply declined to more moderate levels of sensitivity after age 15. It is important to note that in contrast to age-specific predictors that global exposure measures (e.g., duration, multiplicity, severity) were weak predictors of a history of MDD in males.

#### Females

The random forest classification model also showed significant predictive ability in females with accuracy of 0.672 ± 0.059, kappa = 0.216 ± 0.142 (*t*_9_ = 4.80 *p* < 0007), and ROC area of 0.597 ± 0.064 (*t*_9_ = 4.78. *p* < 0.0007). Average fit across models on test and training set together provided specificity of 0.971 (95% CI: 0.938–0.989), sensitivity of 0.467 (95% CI: 0.375–0.560), and kappa = 0.487 (95% CI: 0.391–0.582). Altogether, subjects predicted to have MDD according to the model had a 29.2-fold (95% CI: 12.0–70.9) increase in odds of actually having a history of MDD.

**Figure 4:**
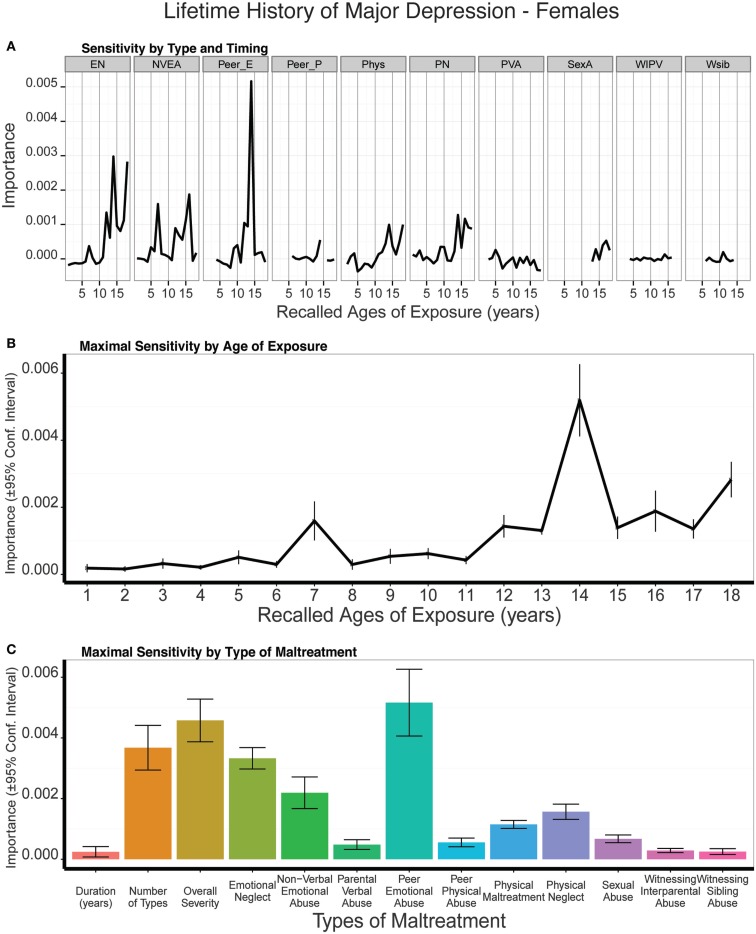
**(A)** Mean importance of age of exposure for each type of maltreatment in predicting history of major depressive disorder in females. Values are missing for ages of exposure for some types of maltreatment if <5% of subjects reported exposure at that age. **(B)** Maximal importance of age of exposure (regardless of type) and **(C)** maximal importance of type of maltreatment (regardless of age) in predicting history of major depressive disorder in females. See Figure 3 for abbreviations.

The single most important predictor in females was peer emotional abuse at age 14, which was a more important predictor than duration (*t*_9_ = 9.29, *p* < 10^-5^), multiplicity (*t*_9_ = 5.78, *p* < 10^-4^), or severity (*t*_9_ = 3.44, *p* < 0.008) (Figure 4). Emotional neglect was the second most important exposure type for females, and NVEA was the third. The composite sensitivity profile showed a transient increase in sensitivity at age 7 and a sustained increase in sensitivity from age 12 on, with an abrupt peak at age 14. While peer emotional abuse at age 14 was the strongest predictor, the global exposure measures were also prominent predictors in females, but not in males.

It is interesting that NVEA was the most important predictor in males and peer emotional abuse in females, as this is opposite to gender differences in exposure patterns. Males, on average, report higher levels of exposure to peer emotional abuse at age 14 than females (*t*_433_ = 2.63, *p* < 0.009), whereas females reported higher levels of exposure to NVEA than males at the same age (*t*_503.7_ = −2.44, *p* < 0.02). Hence, gender differences in sensitivity are not tied to gender differences in overall rates of exposure.

#### Maltreated subjects only

The presence of non-maltreated subject in the sample with and without histories of MDD may skew the results. Indeed, it is possible that type and timing of maltreatment would matter less, or not at all, in a group of subjects who were all exposure to at least one type of maltreatment. However, limiting the analysis to include only subjects exposed to one or more types of maltreatment yielded very similar findings. NVEA at age 14 was the most important predictor in males, and was consistently a much more important predictor across models than duration (*t*_9_ = 21.91, *p* < 10^-8^), multiplicity (*t*_9_ = 17.78 *p* < 10^-7^), or severity (*t*_9_ = 18.53, *p* < 10^-7^). Odds of receiving a lifetime diagnosis of MDD were 19.01-fold (95% CI: 7.60–47.56) greater in maltreated males designated as “at risk” by the model versus maltreated males not at risk. Peer emotional abuse at age 14 was the most important predictor in maltreated females, and was a consistently more important predictor than duration (*t*_9_ = 16.01, *p* < 10^-7^), multiplicity (*t*_9_ = 11.71, *p* < 10^-6^), or severity (*t*_9_ = 11.35, *p* < 10^-5^). Odds of receiving a lifetime diagnosis of MDD were 18.95-fold (95% CI: 8.95–38.70) greater in maltreated females designated as “at risk” by the model versus maltreated females not so designated. Global exposure measures were relatively weak predictors in both maltreated males and maltreated females.

### Relationship between multiplicity of exposure and sensitive exposure periods

**Figure 5:**
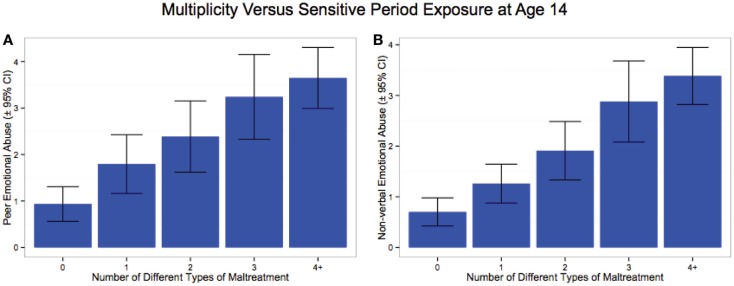
**Increase in severity of exposure at age 14 to (A) peer emotional abuse in females, and (B) non-verbal emotional abuse in males as a function of number of different types of maltreatment experienced across childhood**.

We have hypothesized that a powerful alternative explanation for the appearance of a monotonically increasing relationship between number of different types of childhood maltreatment experienced and risk for depression may be a byproduct of that fact that exposure to more types of maltreatment increases likelihood of experiencing a critical type of maltreatment at a critical age. Hence, we assessed whether multiplicity of exposure was associated with a monotonic increase in severity of exposure to non-verbal emotional abuse at age 14 in males and to peer emotional abuse at age 14 in females. As illustrated in Figure 5 there was indeed a dramatic linear increase in severity of exposure to NVEA in males (F_1,221_ = 101.88, *p* < 10^-16^) and to peer emotional abuse in females (F_1,335_ = 49.15, *p* < 10^-10^) at age 14, with each increment in exposure to different types of maltreatment across childhood.

**Figure 6:**
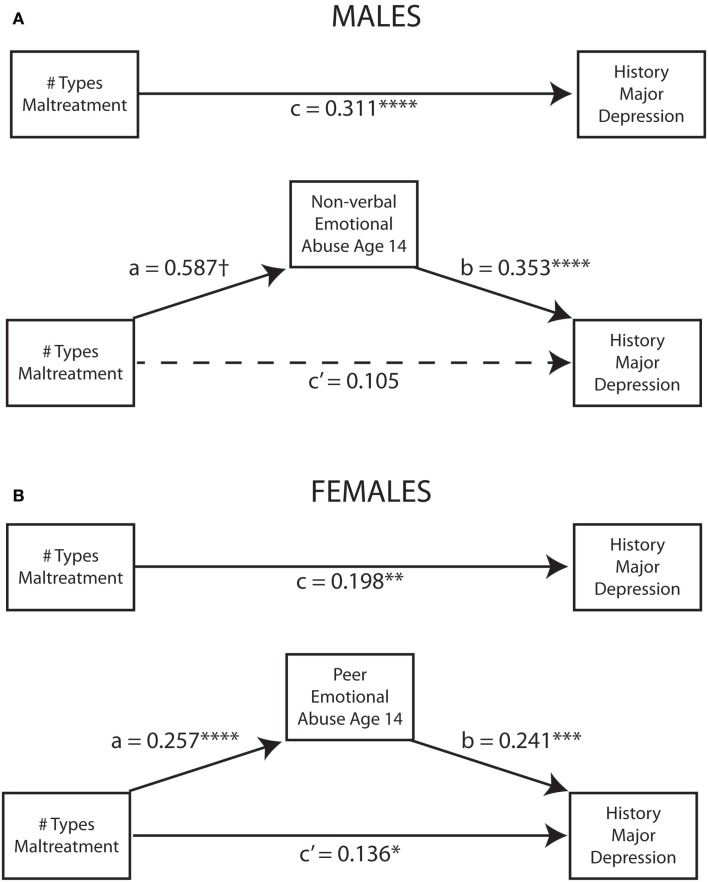
**Mediation models indicating the degree to which (A) non-verbal abuse at age 14 in males and (B) peer emotional abuse at age 14 in females mediated the relationship between number of different types of childhood maltreatment and history of major depressive disorder**. *p < 0.05,**p < 0.01, ***p < 0.001, ****p < 0.0001, ^†^*p* < 10^-16.^

Figure 6 indicates the degree to which NVEA and peer emotional abuse at age 14 in males and females, respectively, mediated the association between number of different types of maltreatment experienced during childhood and risk for major depression. The relationship was strongly mediated in males such that the direct effect between multiplicity and history of major depression was no longer statistically significant once the mediator was taken into account. Overall, the indirect effect in males was substantial (*ab* = 0.207; 95% CI: 0.117–0.312) and 1.98-fold greater than the direct effect. In contrast, the relationship between multiplicity of exposure and risk for major depression in females was only partially mediated by peer emotional abuse at age 14. The direct effect fell from *c* = 0.198 (*p* < 10^-5^) to *c’* = 0.136 (*p* < 0.002) after taking the mediator into account. The indirect effect was modest but significant (*ab* = 0.071, 95% CI: 0.029–0.123, *p* < 0.002) and 38.7% as large as the direct effect. However, the overall statistical association between peer emotional abuse at age 14 and MDD was larger than the direct effect of multiplicity. This analysis also provides confirmation, using a more traditional statistical approach, of the relative importance of NVEA and peer emotional abuse at age 14 as sensitive period risk factors for major depression.

### Type and timing of maltreatment and interviewer-based ratings of depression on the Hamilton depression rating scale

#### Males

**Figure 7:**
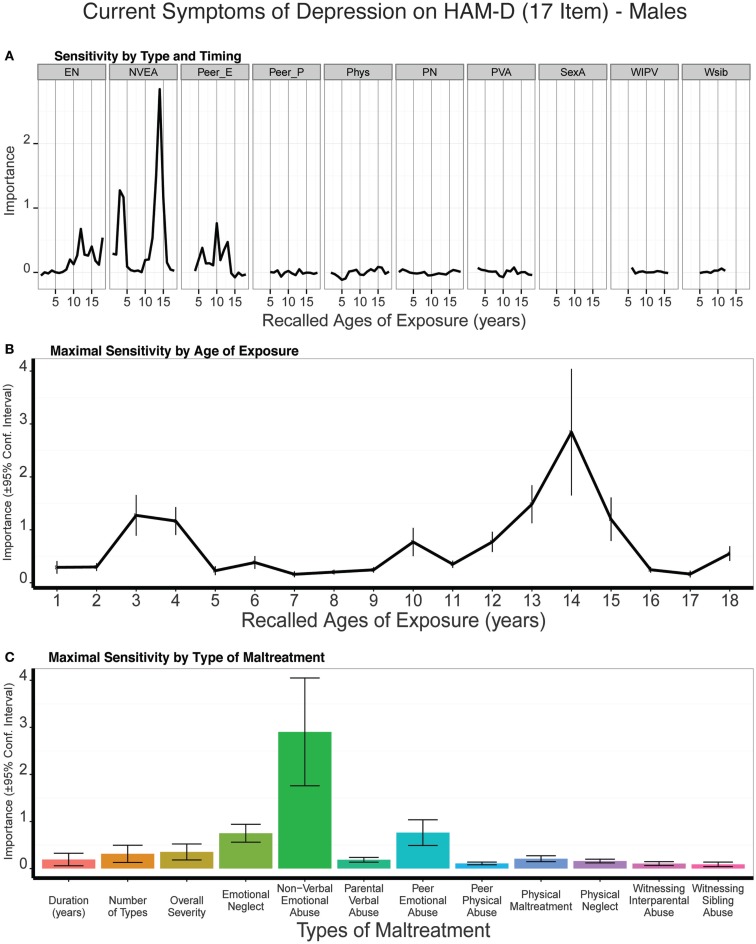
**(A)** Mean importance of age of exposure for each type of maltreatment in predicting symptoms of depression on the 17-item Hamilton Depression Rating Scale in males. Values are missing for ages of exposure for some types of maltreatment if <5% of subjects reported exposure at that age. **(B)** Maximal importance of age of exposure (regardless of type) and **(C)** maximal importance of type of maltreatment (regardless of age) in predicting symptom scores. See Figure 3 for abbreviations.

Predictive modeling of 17-item HDRS scores showed that NVEA at 14 years of age was the strongest single predictor in males, and this was a consistently more important predictor than the three global exposure measures (duration: *t*_9_ = 4.45, *p* < 0.002; multiplicity: *t*_9_ = 4.12, *p* < 0.003; severity: *t*_9_ = 3.39, *p* = 0.008) (Figure 7). Prominent sensitivity to NVEA also emerged at 3–4 years of age. There were modest secondary periods of sensitivity to emotional neglect at age 12 and peer emotional abuse at age 10. Overall, sensitivity was greatest between 13–15 and 3–4 years of age. On average, trained models predicted 9% of the variance in 17-item HDRS scores (*t*_9_ = 4.29 *p* < 0.002) in the test sets.

Mediation analysis indicated that NVEA at age 14 substantially mediated the association between multiplicity of exposure and HDRS-17 scores such that the total relation (*c* = 0.287, *p* < 10^-4^) was rendered non-significant (*c’* = 0.105, *p* < 0.20). The indirect effect (*ab* = 0.182, 95% CI: 0.054–0.331, *p* < 0.006) was 73% greater than the direct effect, and the overall association between NVEA-14 and HDRS-17 was strong (*b* = 0.299, *p* = 0.002).

#### Females

**Figure 8:**
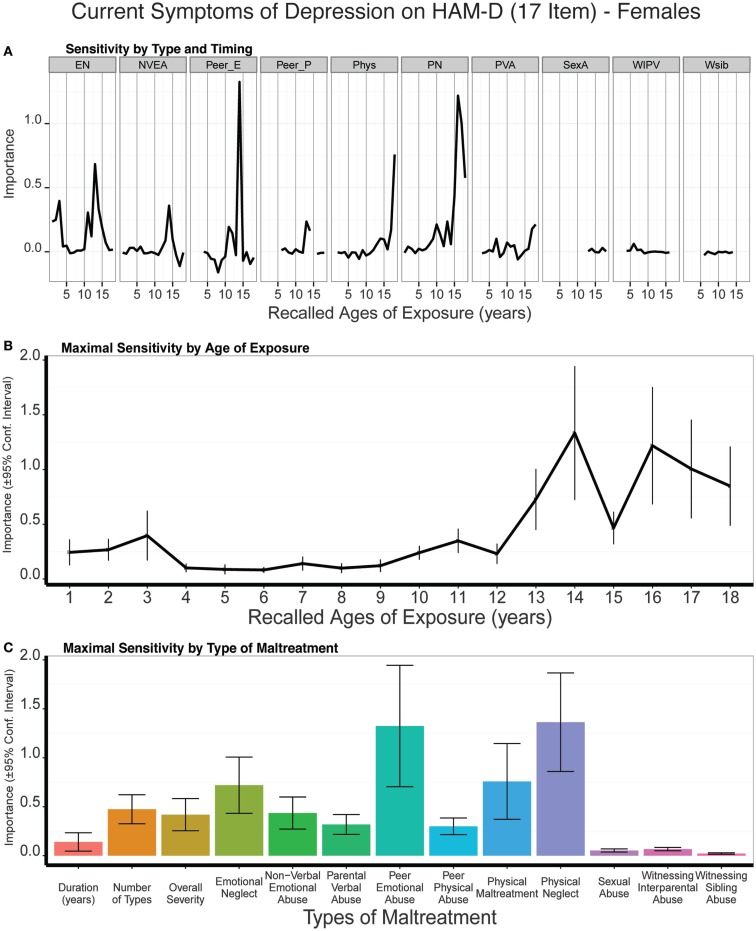
**(A)** Mean importance of age of exposure for each type of maltreatment in predicting symptoms of depression on the 17-item Hamilton Depression Rating Scale in females. Values are missing for ages of exposure for some types of maltreatment if <5% of subjects reported exposure at that age. **(B)** Maximal importance of age of exposure (regardless of type) and **(C)** maximal importance of type of maltreatment (regardless of age) in predicting symptom scores. See Figure 3 for abbreviations.

The strongest single predictor of 17-item HDRS scores in females was peer emotional abuse at age 14, which was a consistently more important predictor than the three global exposure measures (duration: *t*_9_ = 3.27, *p* < 0.01; multiplicity: *t*_9_ = 3.55, *p* < 0.007; severity: *t*_9_ = 2.60, *p* < 0.03) (Figure 8). Physical neglect at age 16, emotional neglect at age 13, and physical maltreatment at age 18 were also relatively important predictors. Together these factors produced a broad band of increased sensitivity between 13 and 18 years of age. There was also a hint of an early peak in sensitivity to emotional neglect at age 3. On average, trained models predicted 7% of the variance in 17-item HDRS scores (*t*_9_ = 3.7 *p* < 0.004) in the test sets. Peer emotional abuse at age 14 did not significantly mediate the association between number of types of childhood maltreatment and HDRS-17 scores (*ab* = 0.047, 95% CI: -0.007 to 0.120, *p* < 0.09).

### Type and timing of maltreatment and interviewer-based ratings of atypical depressive symptoms

#### Males

**Figure 9:**
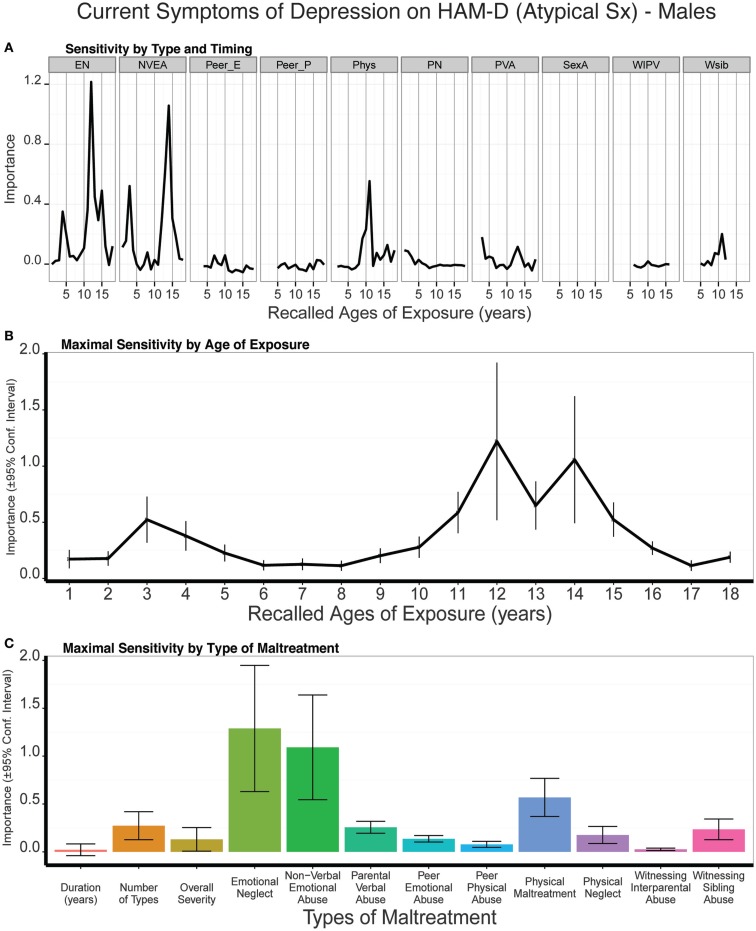
**(A)** Mean importance of age of exposure for each type of maltreatment in predicting symptoms of atypical depression in males. Values are missing for ages of exposure for some types of maltreatment if <5% of subjects reported exposure at that age. **(B)** Maximal importance of age of exposure (regardless of type) and **(C)** maximal importance of type of maltreatment (regardless of age) in predicting symptom scores. See Figure 3 for abbreviations.

Predictive modeling of atypical depression scores in males showed that emotional neglect at 12 years of age was the strongest single predictor whereas exposure to NVEA at 13–14 years of age was the strongest adjacent age predictors (Figure 9). Both emotional neglect at 12 years (all *p* values < 0.03) and NVEA at 13–14 (all *p* values < 0.003) were consistently more important predictors than global exposure measures across the different cross-validated samplings. Overall, atypical rating scores appear to be particularly susceptible to maltreatment at 11–15 years of age. A smaller peak at 3 years of age was also apparent. On average, trained models predicted 10% of the variance in atypical depression scores (*t*_9_ = 3.95 *p* < 0.003) in the test sets.

#### Females

**Figure 10:**
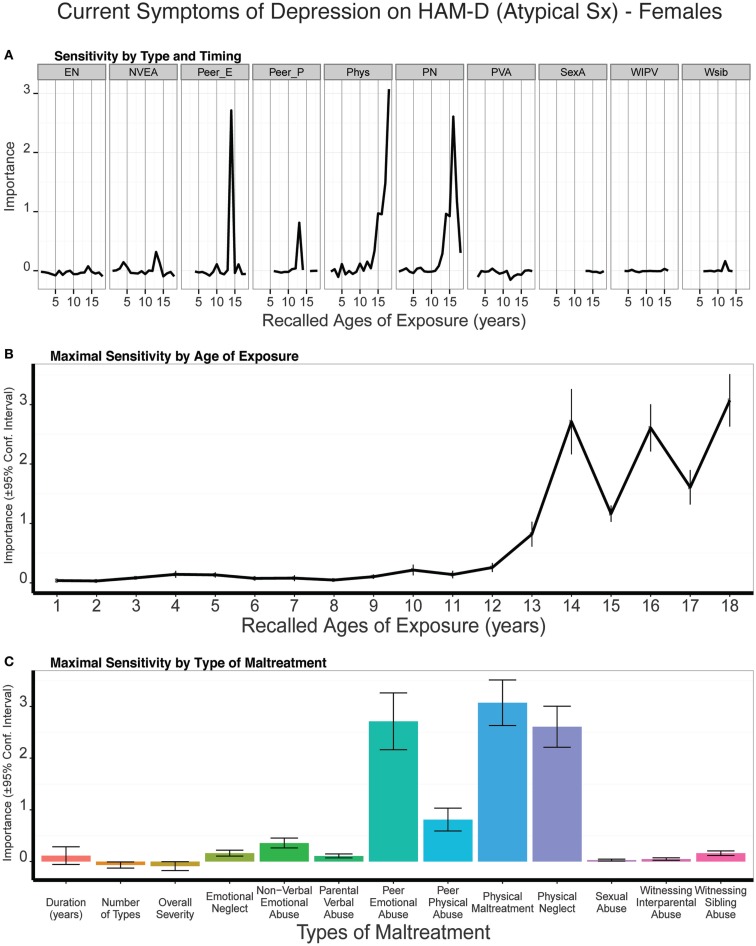
**(A)** Mean importance of age of exposure for each type of maltreatment in predicting symptoms of atypical depression in females. Values are missing for ages of exposure for some types of maltreatment if <5% of subjects reported exposure at that age. **(B)** Maximal importance of age of exposure (regardless of type) and **(C)** maximal importance of type of maltreatment (regardless of age) in predicting symptom scores. See Figure 3 for abbreviations.

Atypical depression scores in females appeared to be most susceptible to exposure to peer emotional abuse at age 14, physical neglect at age 16, and physical abuse at age 18. As seen in Figure 10, all three peak exposure periods were vastly more important predictors of atypical scores than the global exposure measures (e.g., all *p* values < 10^-7^ for importance of physical maltreatment at age 18 versus duration, multiplicity, or severity of exposure). Together, females appeared to be particularly susceptible from age 13 to 18 to one or more of these forms of maltreatment. On average, trained models predicted 6% of the variance in atypical depression scores (*t*_9_ = 3.89 *p* < 0.003) in the test sets.

### Type and timing of maltreatment and current self-reported symptoms of depression on the Symptom Checklist-90

#### Males

**Figure 11:**
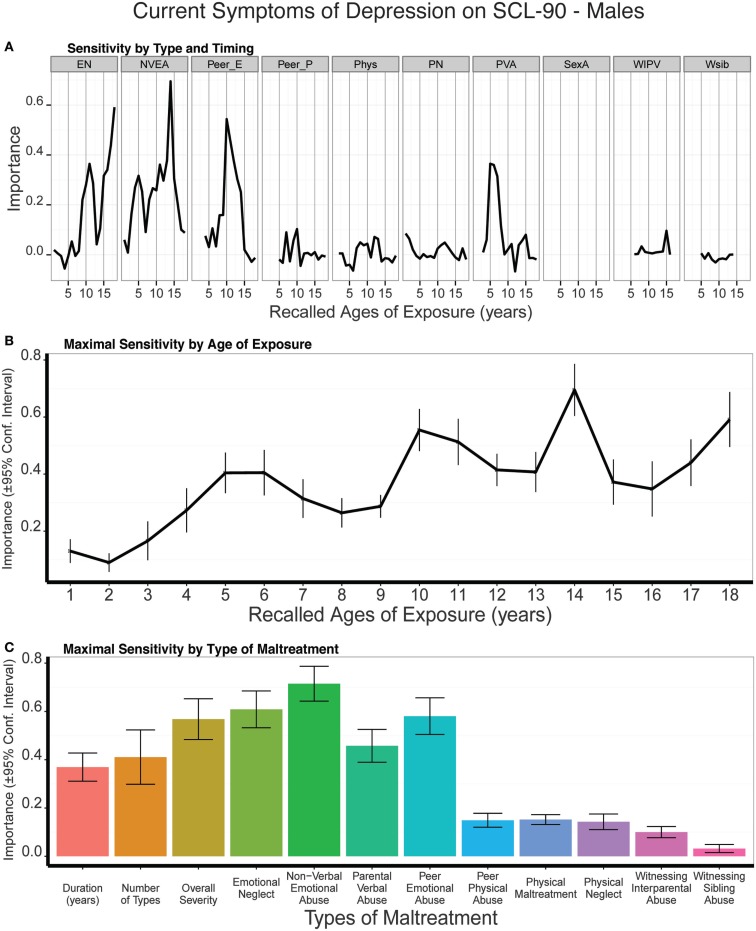
**(A)** Mean importance of age of exposure for each type of maltreatment in predicting current symptoms of depression on the SCL-90 in males. Values are missing for ages of exposure for some types of maltreatment if <5% of subjects reported exposure at that age. **(B)** Maximal importance of age of exposure (regardless of type) and **(C)** maximal importance of type of maltreatment (regardless of age) in predicting symptom scores. See Figure 3 for abbreviations.

As seen in Figure 11, there was slight but significant evidence for a peak in sensitivity to exposure to NVEA at age 14 on current symptom ratings on the SCL-90. Exposure at that age was a more important predictor than three global exposure measures (duration: *t*_9_ = 6.93 *p* < 10^-5^; multiplicity: *t*_9_ = 4.94 *p* < 0.0007; severity: *t*_9_ = 3.17 *p* < 0.011). However, emotional neglect at ages 12 and 18, peer emotional abuse at age 10, and parental verbal abuse at ages 5–6 were also relatively important predictors, which resulted in a broad band of increased sensitivity from age four on. Hence, we do not observe the relatively narrow period of increased sensitivity on this instrument as we had observed in relation to categorical diagnosis or HDRS symptom scores in males. On average, trained models predicted 12% of the variance in SQ depression scores in the test sets (*t*_9_ = 5.13, *p* < 0.0004).

#### Females

**Figure 12:**
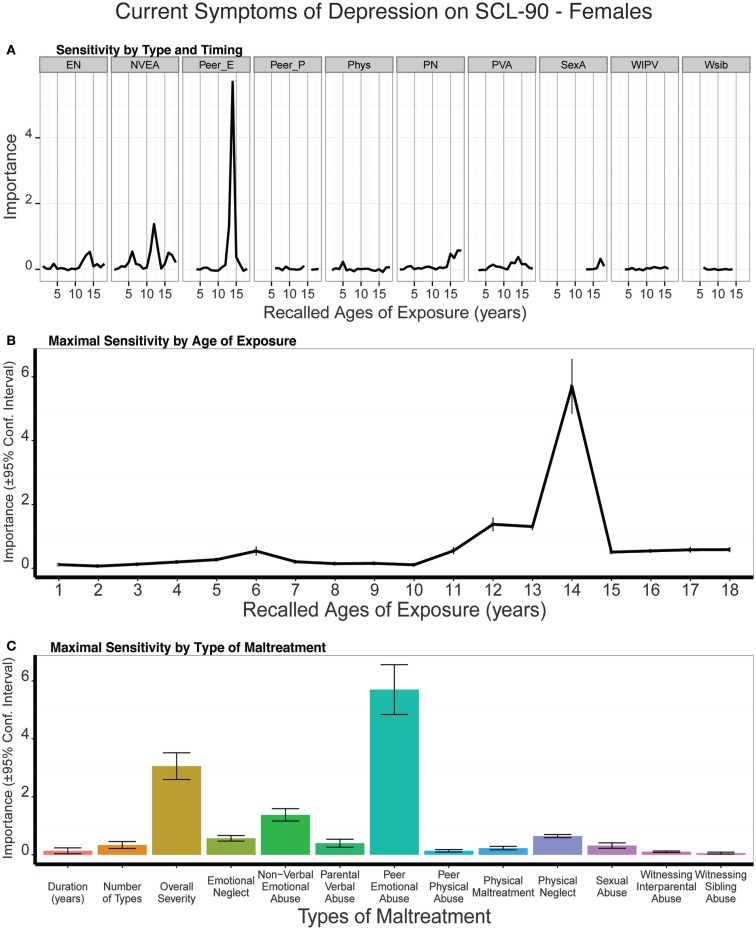
**(A)** Mean importance of age of exposure for each type of maltreatment in predicting current symptoms of depression on the SCL-90 in females. Values are missing for ages of exposure for some types of maltreatment if <5% of subjects reported exposure at that age. **(B)** Maximal importance of age of exposure (regardless of type) and **(C)** maximal importance of type of maltreatment (regardless of age) in predicting symptom scores. See Figure 3 for abbreviations.

Exposure to peer emotional abuse at age 14 was a very strong predictor of SCL-90 depression scores in females, and this measure eclipsed the importance of all three global exposure measures (duration: *t*_9_ = 13.90 *p* < 10^-7^; multiplicity: *t*_9_ = 12.86 *p* < 10^-7^; severity: *t*_9_ = 13.90 *p* < 10^-7^) (Figure 12). Results were quite different in females than males, as females showed a transient increase in sensitivity at age six and a very narrow band of increased sensitivity to maltreatment at 12–14 years of age. On average, the trained conditional forest models predicted 14% of the variance in SCL-90 depression scores in the test sets (*t*_9_ = 9.19, *p* < 10^-6^).

### Type and timing of maltreatment and current self-reported symptoms of depression – Kellner symptom questionnaire

#### Males

As seen in Figure S1 in Supplementary Material, the single most important predictor of SQ depression scores in males was exposure to parental verbal abuse at 12 years of age. This was a more important predictor than overall duration or multiplicity of exposure but was not a significantly more important predictor than severity (*t*_9_ = -0.18, *p* > 0.8). However, exposure to parental verbal abuse at 11–12 years of age was a more important predictor than all three measures of global exposure including severity (*t*_9_ = 5.47, *p* < 0.0004), satisfying our minimum *a priori* requirement for presence of a sensitive exposure period. However, in addition to this peak, there was an extended period of sensitivity to NVEA from 3 to 15 years of age, resulting in a moderate elevation in sensitivity from age 3 on. Hence, there was no evidence for a narrow band of sensitivity for depression scores on this instrument in males. Overall, trained conditional forest models predicted, on average, 14% of the variance in SQ depression scores in the test sets (*t*_9_ = 6.00, *p* = 0.0001).

#### Females

Peer emotional abuse at age 14 was the most important predictor of current SQ depression scores in females (Figure S2 in Supplementary Material). Exposure to this specific type of abuse at this single time point eclipsed the importance of all three global exposure measures (duration: *t*_9_ = 29.66 *p* < 10^-9^; multiplicity: *t*_9_ = 23.91 *p* < 10^-8^; severity: *t*_9_ = 21.15 *p* < 10^-8^), and resulted in the presence of a narrow band of sensitivity from 11 to 14 years of age, with the added presence of a minor band of increased sensitivity at 5–6 years of age. Trained conditional forest models predicted, on average, 17% of the variance in SQ depression scores in the test sets (*t*_9_ = 6.47, *p* < 10^-4^).

### Type and timing of maltreatment and self-reported suicidal ideation

#### Males

**Figure 13:**
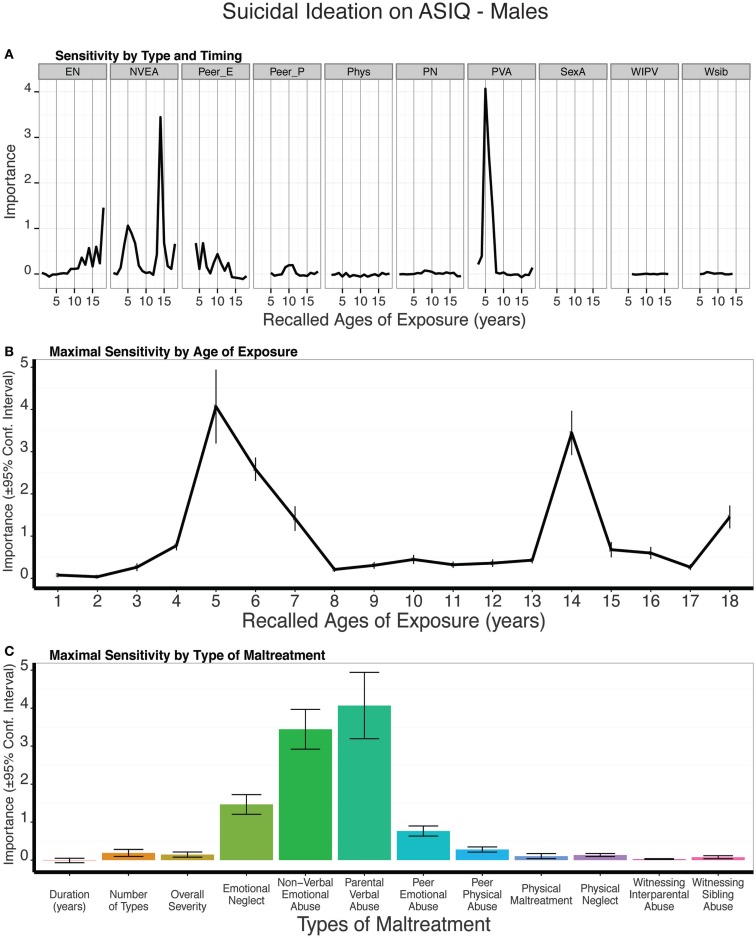
**(A)** Mean importance of age of exposure for each type of maltreatment in predicting suicidal ideation on the Adult Suicidal Ideation Questionnaire in males. Values are missing for ages of exposure for some types of maltreatment if <5% of subjects reported exposure at that age. **(B)** Maximal importance of age of exposure (regardless of type) and **(C)** maximal importance of type of maltreatment (regardless of age) in predicting symptom scores. See Figure 3 for abbreviations.

As seen in Figure 13, predictive modeling of suicidal ideation in males revealed prominent importance of NVEA at age 14 and parental verbal abuse at age 5, which was a substantially more important predictor than all three global exposure measures (duration: *t*_9_ = 10.99, *p* < 10^-5^; multiplicity: *t*_9_ = 10.52 *p* < 10^-5^; severity: *t*_9_ = 9.06 *p* < 10^-5^). The composite sensitivity profile revealed two bands of markedly increased sensitivity at 5–7 years of age, and at age 14. On average, the trained models predicted 17% of the variance in ASIQ scores (*t*_9_ = 5.22, *p* < 0.0004) in the test sets.

#### Females

**Figure 14:**
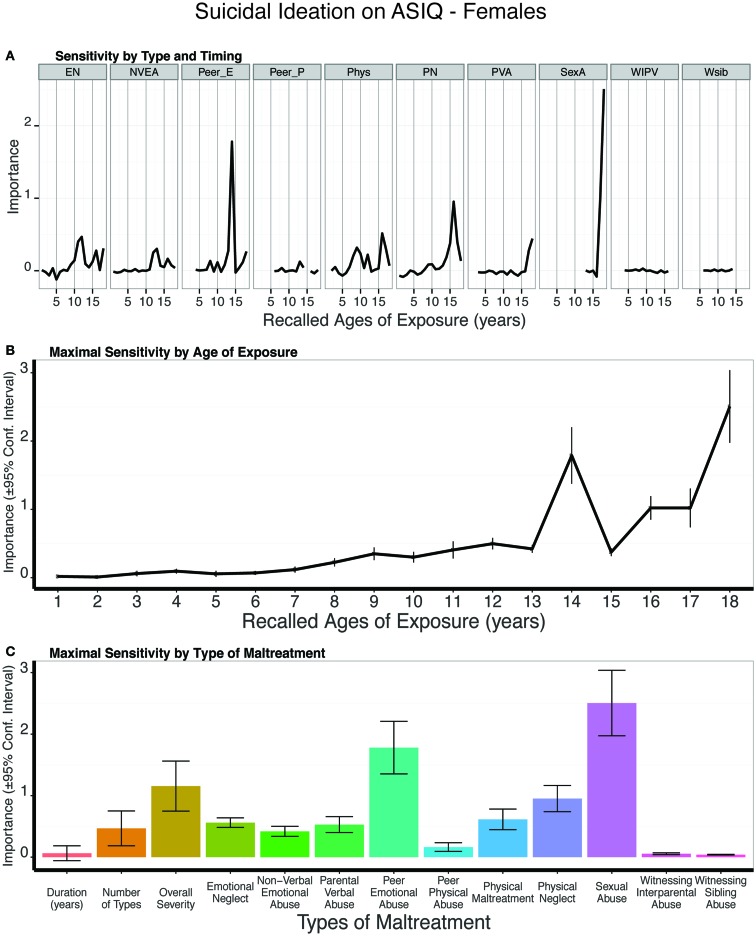
**(A)** Mean importance of age of exposure for each type of maltreatment in predicting suicidal ideation on the Adult Suicidal Ideation Questionnaire in females. Values are missing for ages of exposure for some types of maltreatment if <5% of subjects reported exposure at that age. **(B)** Maximal importance of age of exposure (regardless of type) and **(C)** maximal importance of type of maltreatment (regardless of age) in predicting symptom scores. See Figure 3 for abbreviations.

Predictive modeling of suicidal ideation ratings in females showed that peer emotional abuse at 14 years of age remained an important predictor (Figure 14). However, the single most important predictor was sexual abuse at 18 years. This was a significantly more important predictor than all three global exposure measures (duration: t_9_ = 4.67, *p* = 0.001; multiplicity: t_9_ = 3.99 *p* = 0.003; severity: t_9_ = 2.48 *p* < 0.04). The composite sensitivity profile revealed a prominent increase in sensitivity at age 14 and from age 16 on. On average, the trained models predicted 9% of the variance in ASIQ scores (t_9_ = 4.56, *p* < 0.001) in the test sets.

### Type and timing of maltreatment and symptoms of limbic irritability

As seen in Figures S3 and S4 in Supplementary Material, the most important predictors of LSCL-33 scores were global exposure measures, particularly number of different types of maltreatment and severity of maltreatment experienced across childhood.

#### Males

Both multiplicity (*t*_9_ = -3.94, *p* < 0.004) and severity (t_9_ = -3.62, *p* < 0.006) of exposure across childhood were more important predictors than exposure to parental verbal abuse at age 15 (the most important single age predictor) (Figure S3 in Supplementary Material). Further, these global exposure measures were as important as exposure to parental verbal abuse at ages 14–15, which was the most important adjacent age predictor. Hence, by our definitions, we cannot reject the null hypothesis, and do not find evidence for a sensitive exposure period on ratings of limbic irritability in males. On average, the trained models predicted 25% of the variance in LSCL33 scores (*t*_9_ = 6.88, *p* < 10^-4^) in the test sets.

#### Females

The importance of global exposure measures was even more salient in females. Multiplicity of exposure (*t*_9_ = -14.79, *p* < 10^-6^) and severity of exposure across childhood (*t*_9_ = 35.62, *p* < 10^-10^) were much more important predictors than the most salient single age predictor (peer emotional abuse at age 13), and were also much more important predictors (both *p* < 10^-5^) than the most salient adjacent age predictor (peer emotional abuse at 13–14 years) (Figure S4 in Supplementary Material). In short, there was no evidence for a significant sensitive exposure period effect on ratings of limbic irritability in females. On average, the trained models predicted 21% of the variance in LSCL33 scores (*t*_9_ = 7.08, *p* < 10^-4^) in the test sets.

## Discussion

These findings provide strong support for the presence of brief modality-specific sensitive exposure periods when maltreatment appears to exert maximal impact on risk for a diagnosis of major depression, current symptoms of depression, and degree of suicidal ideation. Males and females appeared to be particularly sensitive to exposure at 14 years of age, though females were particularly sensitive to peer emotional abuse, while males were most vulnerable to parental non-verbal emotional abuse at this age.

Exposure to peer emotional abuse at age 14 was the most important predictor in females of a history of MDD and symptoms of depression on the SQ, SCL90, and HDRS-17. In contrast, non-verbal emotional abuse at age 14 was the most important predictor in males of history of MDD and severity of depression on the HDRS-17, and strongly mediated the association between multiplicity of exposure and outcome. NVEA is characterized by a parent or other important parental figure: (1) being very difficult to please; (2) not having the time or interest to talk to you; 3) withholding important secrets; and 4) causing you to prematurely shoulder adult responsibilities. In a sense, NVEA is a form of parental rejection, while peer emotional abuse and ostracism is a form of peer rejection. Hence, the present findings suggest that being rejected at about 14 years of age may be a crucial underlying risk factor for the emergence of depression in both males and females.

The idea that rejection during adolescence leads to depression is not new. Prominent associations between rejection and depression have emerged in both cross-sectional (102, 103) and longitudinal studies (102, 104–108). However, the relationship may be complex and bidirectional with rejection leading to depression and depression leading to rejection (109). In one of the better studies, Nolan et al. (105) followed 240 adolescents for 3 years and found through a cross-lagged structural equation model that rejection at one time point predicted depression at a subsequent time, but they did not find the reverse to be true. Prospective studies have also reported gender differences (105, 106, 108) showing that the depressogenic consequences of peer rejection were stronger in females than males (106, 108). This is consistent with our observation that peer emotional abuse and ostracism was a much more important predictor in females than males.

Interestingly, the neural substrates for pain associated with social rejection overlap extensively with neural substrates for physical pain (110). In particular, degree of distress engendered by rejection correlates with degree of activation of the anterior insula (110). We have recently reported that the right anterior insula appears to be a much more important cortical network hub in maltreatment individuals than in unexposed controls (63). Hence, maltreated individuals may feel or respond to social rejection to an even greater degree than unexposed controls.

Peer emotional abuse and non-verbal emotional abuse at age 14 were also important predictors of suicidal ideation in females and males, respectively. However, they were not the most important predictors. The most important predictor in females was sexual abuse at age 18 and parental verbal abuse at age 5 in males. These findings are consistent with the idea that the vast majority of individuals attempting suicide are depressed, but that the majority of depressed individuals never attempt suicide. Hence, other factors likely come into play besides depression in determining risk for suicide or degree of suicidal ideation. Several studies have reported robust associations between sexual abuse, suicidal ideation, and suicide attempts (111–119). Exposure to sexual abuse at age 18 in our sample was often a result of dating violence, which has been shown to be independently associated with suicidal ideation and attempts (120–123). Feeling rejected by peers in adolescence followed by dating violence at 18 years may lead to profound feelings of isolation and social rejection in females.

The influence of parental verbal abuse at 5 years of age on suicidal ideation in 18- to 25-year-old males is more difficult to understand. In psychodynamic psychotherapy, this corresponds to the transition between phallic and latency stage and is associated with resolution of the Oedipal Conflict, as illustrated by the case of the 5-year-old boy “Little Hans” (124). In this imaginative theory, boys are in love with their mothers but fearful of reprisal by their fathers. They resolve this conflict by identifying with the father and taking on a male gender role. As fanciful as this theory seems, it may be the case that boys are acutely sensitive to criticism by their parents at this stage. Indeed, in Bowlby’s reinterpretation of “Little Hans”, he stresses the importance of a “secure base” and risk for developing an anxious attachment in its absence (125). Insecure attachments, particularly avoidant, are a significant risk factor for suicidal behavior (126).

Delineating sensitive exposure periods to specific types of maltreatment may aid in the analysis of gender specific differences in experience associated with suicidal thoughts and behavior. It would be interesting and informative to collect MACE exposure data on groups of individuals who have made severe or near-successful suicide attempts.

Emotional neglect at age 12 was typically the second most important predictor variable in both males and females in determining risk for MDD and symptom severity on the 17-item HDRS and Atypical Index. Emotional neglect on the MACE was determined by response to questions indicating whether mother or father figures were present in the home, but emotionally unavailable for a variety of reasons like drugs, alcohol, being a workaholic or having an affair, plus reverse scores on items indicating whether family member made you feel loved, family member helped you feel special or important, and whether family was a source of strength and support. We wonder if being more emotionally nurtured at age 12 would protect against the impact of emotional abuse at age 14.

The random forest regression findings indicating the importance of non-verbal emotional abuse, peer emotional abuse, and emotional neglect at specific ages are also consistent with the result of the linear mixed effect model analyses (Figure 2). The mixed model analyses showed that maltreated subjects with versus without histories of depression differed most significant in their time course of exposure to non-verbal emotional abuse, peer emotional abuse, and emotional neglect.

The shared vulnerability of males and females to emotional neglect at age 12 and parallel vulnerability at age 14 to peer emotional abuse in females and parental NVEA in males is remarkable. We find this cross-gender temporal consistency in vulnerability to be a compelling outcome of these analyses.

Although these periadolescent peaks were the most prominent, it is important to note that there were some additional periods of increased sensitivity, typically at 3–4 or 5–7 years of age. For example, exposure to NVEA at 3–4 years of age was a significant predictor in males for HDRS and Atypical Index scores, as was emotional neglect at age 3 for HDRS ratings in females. Exposure to parental verbal abuse at 5–6 years was the most important predictors of suicidal ideation scores in males and a significant predictor of SQ depression scores in females. We found through simulation studies that the conditional forest model is very conservative and that even relatively low amplitude peaks that consistently emerge above background are more likely real (tied to outcomes) than artifactual. The idea that there are both early and late periods of sensitivity fits with our observation that the hippocampus has both early and late (pubertal) periods of increased sensitivity to maltreatment (43, 45), and we have hypothesized that brain regions may be vulnerable both during early stage of synaptic and dendritic overproduction and again during the pruning process (1). Soumi et al. has also found evidence for two sensitive periods in his primate model of maltreatment, with the second phase occurring during the peripubertal period.

Sensitive exposure periods identified in this manner may overlap with previously identified developmental stages and provide new insight into their nature. Further, measures of gray matter volume and fiber tract integrity appear to have similar discrete sensitive exposure periods (41, 45). Hence, the search for sensitive exposure periods may provide a new way of linking developmental psychopathology with developmental neurobiology.

It is also worth noting that presence of sensitive exposure periods for current symptoms of depression on the SQ and SCL90 were more apparent in females than males. Males, however, showed strong evidence for sensitive exposure periods on SCID, HDRS-17, and ASIQ. Major depression is characterized by an array of symptoms and different instruments emphasize different components of the disorder. The HDRS, for instance, was developed for use with hospitalized patience and emphasizes melancholic and physical symptoms. Different instruments also assess different time frames (e.g., lifetime diagnosis, monthly suicidal ideation ratings, or weekly SQ and SCL90 scores). Hence, it may be the case that sensitive exposure periods are more discernible on some instruments than others as they emphasize a different array or symptoms or cover a longer assessment period, perhaps resulting in a more salient signal.

The next step in exploring the importance of sensitive exposure periods and depression may be to focus on specific domains of function related to the disorder such as anhedonia or rejection sensitivity. Some of these functions may correspond to specific Research Domain Criteria and related circuits (127).

It is important to emphasize that not every disorder or set of symptoms associated with childhood maltreatment will necessarily manifest a sensitive exposure period. This is clearly the case in terms of “limbic irritability”„ as overall severity of exposure was a much more important predictor than exposure to a specific type of maltreatment at one or two adjacent ages. However, the observation that some disorders have narrow sensitive exposure periods has far reaching implications.

First, it provides a vastly different way of explaining the ACE study finding of an essentially linear increase in risk or consequence of exposure to maltreatment based on number of different types of early adversity experienced. The ACE score has been framed by its creators as *“a measure of the cumulative exposure to traumatic stress during childhood”* (128), and they emphasize the importance of cumulative burden and downplay the importance of exposure to specific types of maltreatment (128).

We hypothesized that the linear increase associated with exposure to number of different types of maltreatment may be a statistical byproduct of the fact that exposure to more types of abuse increases the chance of experiencing a critically deleterious form of maltreatment at a critical age. In support of this alternative hypothesis, we found that exposure to a specific type of maltreatment during a narrow developmental stage was a more important predictor of depression-related outcomes than overall measures of exposure as indexed by severity, duration, or multiplicity. Hence, the present findings provide strong support for this alternative view regarding the association between maltreatment and diagnosis and symptoms of depression or suicidal ideation.

Our findings do not in any way cast doubt on the results of the ACE study, just their interpretation. We found the same graded dose-response relationship between outcome and multiplicity of exposure in our sample as they found in theirs. What our results show, however, is that exposure to a specific type of maltreatment at a specific age was a more important predictor than multiplicity, and that it largely mediated the association, particularly in males.

This distinction is of paramount importance in exploring the mechanisms linking exposure to early life stress and risk for psychopathology. A cumulative burden hypothesis fits with the concept of allostatic load (31, 129) but is relatively non-specific. A sensitive exposure period model, in contrast, is quite specific as it ties the effect to a precise developmental period, and in doing so links the phenomenon with developmental psychology, developmental psychopathology, and developmental neurobiology. Hence, finding that exposure to peer emotional abuse at age 14 is the most important predictor of risk for history of major depression in females, raises questions about the psychological impact of peer rejection at this age, as well as questions about which brain regions and pathways are particularly vulnerable at this stage. Further, a sensitive exposure period perspective may provide novel insights regarding prevention, preemption, and treatment.

One key implication of these finding is to focus awareness on the vulnerability of boys and girls to emotional neglect at age 12 and to emotional maltreatment and rejection by parents or peers at around 14 years of age. Schools have been taking the issue of bullying more seriously in recent years and this is important. It would be interesting to know if strategies to actively foster social acceptance of early teens within their family and peer networks is effective in reducing long-term risk for depression. It would also be important to know if early interventions targeting young teens who experienced peer or parental emotional abuse may be preemptive.

Second, the presence of sensitive exposure periods may lead to a more precise clinical and neurobiological understanding of susceptibility and resilience. We are presently conducting a study, in which we define adults as relatively resilient to development of MDD if they experienced three or more forms of childhood maltreatment but have no lifetime history of MDD. This made sense as exposure to three or more forms of childhood maltreatment increased odds of receiving a lifetime history of MDD in this sample by 4.64-fold (95% CI: 2.82–7.66). However, subjects defined as “at risk” by the model were at 32.45-fold (95% CI: 15.76–66.79) greater risk, indicating that analysis of type and timing of maltreatment may provide a stronger predictor of risk. Further, exposure to three or more forms of childhood maltreatment did not increase odd of receiving a lifetime diagnosis in subjects whose exposure pattern did not place them “at risk” by the model (odds ratio = 1.21; 95% CI: 0.66–2.24). In contrast, the odds ratio was 35.90 (95% CI: 16.07–80.23) for “at risk” individuals with exposure to three or more forms of maltreatment. Hence, it is the subset of individuals with a specific array of experiences that places them “at risk,” that can be truly deemed resilient if they have not developed MDD. This suggests that studies defining resilience by exposure to a certain number of types of maltreatment or by exposure to a specific type of maltreatment (e.g., sexual abuse) may include a substantial proportion of individuals whose susceptibility or resilience is not known as they were not exposed during sensitive exposure periods.

There are a number of limitations to this study. First, the sample was recruited from the community and enriched to have approximately equal cell sizes for exposure to 0, 1, 2, 3, and 4+ forms of maltreatment. However, we did not enrich the sample to have equal exposure to different types of maltreatment across development. On average, exposure levels to peer emotional abuse, parental verbal and non-verbal emotional abuse, and parental physical abuse were substantially higher than exposure levels to other forms of maltreatment. In particular, relatively few participants reported exposure to childhood sexual abuse at younger ages, so that the analyses only included childhood sexual abuse at ages 12–18 in females, and did not include childhood sexual abuse at all in males, as fewer than 5% of males reported experiencing it to any degree during any given year. Hence, the importance of early exposure to childhood sexual abuse is unknown and unknowable in this sample.

We have previously reported, in similar samples, prominent effects of childhood sexual abuse on psychiatric symptom scores (1, 46), risk for depression (130), cognition (131) and neurobiology (43, 84, 132), but did not control for their exposure to all other forms of maltreatment. However, individuals reporting significant exposure to childhood sexual abuse in this sample also reported significant exposure to 4.2 ± 2.4 other forms of maltreatment. We, like others, had originally assumed that childhood sexual abuse was especially toxic and the key determinant of outcome. This is not necessarily so. Indeed, it may be the case that a substantial proportion of the impact of exposure to childhood sexual abuse results from increased risk of exposure to other forms of maltreatment, such as peer emotional abuse, during sensitive exposure periods.

We did find that exposure to sexual abuse at age 18 was the most important predictor of suicidal ideation in females in this sample. Hence, the importance of sexual abuse from 12 to 18 can be detected by these analyses. That it did not emerge as an important predictor of other outcomes suggests either that sexual abuse has less direct impact than expected, or that it is of primary importance when it occurs at earlier ages. Studies using the CTQ and other instruments to assess exposure to multiple forms of abuse have often found that emotional abuse and emotional neglect are much more strongly related to measures of psychopathology than sexual abuse [e.g., Ref. (133–140)].

This is not to say that childhood sexual abuse is inconsequential. What we found in reviewing the literature is that sexual abuse invariably emerges as a significant risk factor for suicide attempts. For example, Wiederman et al. (141) ascertained self-reported histories of childhood abuse and suicide attempts in 151 women presenting for non-emergent medical care. Increased suicide attempt rates were evident among women who had been sexually or physically abused, or had experienced emotional abuse or witnessed violence. In a multivariate analysis, only sexual abuse and physical abuse were uniquely predictive of having attempted suicide. Similarly, Kaslow et al. (142) compared ratings of family functioning between female African-American suicide attempters (*n* = 126) and non-attempters (*n* = 112). In a multivariate logistic regression, only marital discord and childhood sexual abuse emerged as risk factors for suicide attempts. Other studies have reported strong associations between sexual abuse, suicidal ideation, and suicidal behaviors [e.g., Ref. (143–148)]. Hence, our findings are consistent with the possibility that exposure to childhood sexual abuse is a more salient risk factor for suicidal ideation than depression. Nevertheless, further studies will need to include additional subjects with early exposure to childhood sexual abuse to provide accurate answers.

It is also not the case that the random forest with conditional trees analyses simply ascribes importance to the most prevalent types of exposure. As indicated, the strongest predictive factor in females was peer emotional abuse at age 14, whereas non-verbal emotional abuse at age 14 was the strongest predictor in males. However, males reported significantly more exposure to peer emotional abuse at age 14 than females, while females reported significantly more exposure to non-verbal emotional abuse at age 14 than males. Further, prevalence of exposure to parental physical maltreatment and parental verbal abuse were higher than overall prevalence rates for non-verbal emotional abuse and emotional neglect, though the later were much more important predictors of most outcomes. Similarly, sexual abuse at age 18 would not have emerged as an important predictor of suicidal ideation if degree of reported exposure mattered.

A key limitation of this study is reliance on retrospective self-report. Concerns have been expressed about the reliability of retrospective self-report of maltreatment for a number of reasons including the presence of memory impairment associated with psychopathology, and the presence of specific mood-congruent memory biases associated with psychopathology (149). Brewin et al. (149), in their detailed review, found little evidence to support these criticisms. What we know in comparing retrospective to prospective reports is that adults minimize their degree of exposure on retrospective report (150, 151). Hence, retrospective exposure rates are lower than prospective rates suggesting a problem with false negative reports but not false positive reports. Individuals reporting abuse retrospectively were those who typically endured the most severe abuse on prospective assessment (150). This fits with other studies showing that adult reports of abuse are verifiable (152).

Modern instruments for assessing maltreatment including the MACE generally follow Brewin et al. (149) recommendation to focus on the occurrence of specific events rather than attitude toward events, and all show impressive test–retest reliability [e.g., CTQ *r* = 0.88 (64), MACE *r* = 0.91]. On the MACE, there is no evidence of negative attribution bias. Ratings of depression and anxiety together account for <3% of the variance in retest scores, and the results are in the opposite direction. Increased levels of depression and anxiety were associated with slightly lower retest scores. Indeed, self-reported exposure to maltreatment has been found to be highly consistent over years even in psychotic individuals and not significantly influenced by the severity of their psychosis or their depressive symptoms (153). The observation that recollected exposure to different types of maltreatment follow their own relatively unique time frames (Figure 2) clearly indicates that subjects are nuanced in their response and not simply painting their childhoods as positive or negative based on current mood.

Neurobiological studies also provide strong convergent evidence for the veracity of self-report. For example, unbiased whole brain analyses have specifically identified alteration in the visual cortex (41) and visual-limbic pathway (39) in adults reporting witnessing domestic violence, in the auditory cortex (41) and pathways connecting Broca and Wernicke’s area (39) in adults reporting high levels of exposure to parental verbal abuse, and in genital representation area of somatosensory cortex in women reporting childhood sexual abuse (42). Hence, at least at the group level, there is forensic/anatomical evidence supporting the accuracy of self-report regarding type of maltreatment experienced.

Neurobiological evidence is also emerging to support veracity of claims regarding self-reported ages of exposure. For example, we reported that visually witnessing domestic violence affects the integrity of the inferior longitudinal fasciculus which interconnects visual cortex and limbic system and determines memory and emotional response to things we see (39). Diffusion tensor imaging indicates that maltreated and controls differ in degree of myelination and that this is most strongly affected by exposure between 7 and 13 years (39). Independent studies indicate that this pathway rapidly myelinates between 7 and 15 years of age, supporting the apparent sensitivity to exposure within this age range. Similarly, we know that visual cortex is highly plastic in primates until about the time of puberty. Effects of witnessing domestic violence and experiencing sexual abuse on gray matter volume of visual cortex is highly significant prior to (132) or surrounding puberty (41) but not after.

In short, retrospective self-report appears to provide potentially useful data for the initial exploration of sensitive exposure periods. These findings, in turn, should lead to the development of prospective longitudinal studies that assess subjects as they pass through these sensitive exposure periods, providing new insights into the relationship between early life stress and psychopathology.

## Conflict of Interest Statement

The authors declare that the research was conducted in the absence of any commercial or financial relationships that could be construed as a potential conflict of interest.

## Supplementary Material

The Supplementary Material for this article can be found online at http://journal.frontiersin.org/article/10.3389/fpsyt.2015.00042
