# Supplementary material for: Childhood Maltreatment, Depression, and Suicidal Ideation: Critical Importance of Parental and Peer Emotional Abuse during Developmental Sensitive Periods in Males and Females
Source: Front Psychiatry. 2015 Mar 30;6:42. doi: 10.3389/fpsyt.2015.00042 (PMC4378368; doi:10.3389/fpsyt.2015.00042)
Supplement: Supplementary file 4 [file Image_4.PDF]

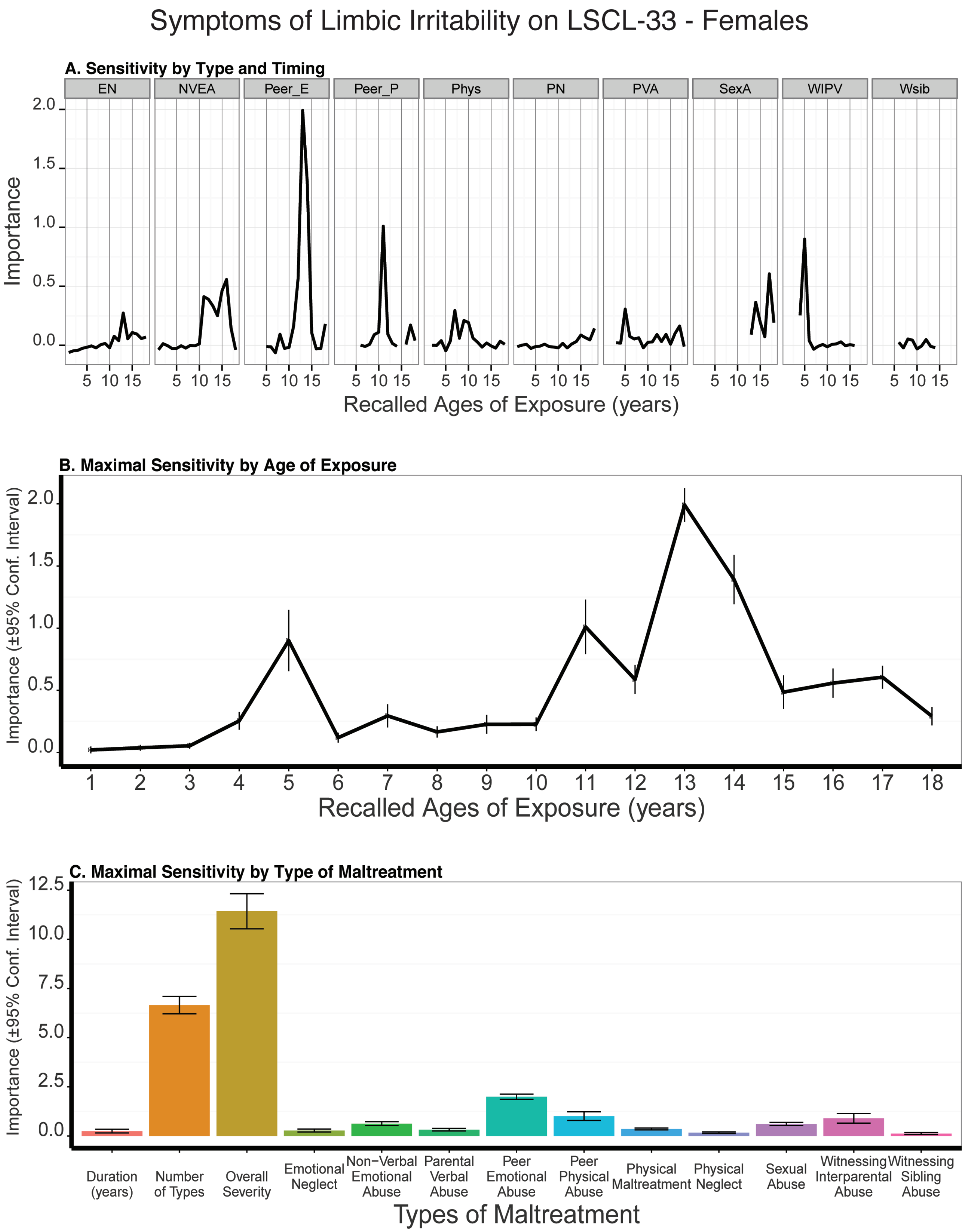

Appendix 4. (A) Mean importance of age of exposure for each type of maltreatment in predicting limbic system 33 scores in females. Values are missing for ages of exposure for some types of maltreatment if less than 5% of subjects reported exposure at that age. **(B)** Maximal importance of age of exposure (regardless of type) and **(C)** maximal importance of type of maltreatment (regardless of age) in predicting symptom scores. See Fig. 3 for abbreviations.
